# *Neisseria gonorrhoeae* MlaA influences gonococcal virulence and membrane vesicle production

**DOI:** 10.1371/journal.ppat.1007385

**Published:** 2019-03-07

**Authors:** Benjamin I. Baarda, Ryszard A. Zielke, Adriana Le Van, Ann E. Jerse, Aleksandra E. Sikora

**Affiliations:** 1 Department of Pharmaceutical Sciences, College of Pharmacy, Oregon State University, Corvallis, Oregon, United States of America; 2 Department of Microbiology and Immunology, F. Edward Hebert School of Medicine, Uniformed Services University of the Health Sciences, Bethesda, Maryland, United States of America; 3 Vaccine and Gene Therapy Institute, Oregon Health & Science University, Beaverton, Oregon, United States of America; University of Oxford, UNITED KINGDOM

## Abstract

The six-component maintenance of lipid asymmetry (Mla) system is responsible for retrograde transport of phospholipids, ensuring the barrier function of the Gram-negative cell envelope. Located within the outer membrane, MlaA (VacJ) acts as a channel to shuttle phospholipids from the outer leaflet. We identified *Neisseria gonorrhoeae* MlaA (*ngo2121*) during high-throughput proteomic mining for potential therapeutic targets against this medically important human pathogen. Our follow-up phenotypic microarrays revealed that lack of MlaA results in a complex sensitivity phenome. Herein we focused on MlaA function in cell envelope biogenesis and pathogenesis. We demonstrate the existence of two MlaA classes among 21 bacterial species, characterized by the presence or lack of a lipoprotein signal peptide. Purified truncated *N*. *gonorrhoeae* MlaA elicited antibodies that cross-reacted with a panel of different *Neisseria*. Little is known about MlaA expression; we provide the first evidence that MlaA levels increase in stationary phase and under anaerobiosis but decrease during iron starvation. Lack of MlaA resulted in higher cell counts during conditions mimicking different host niches; however, it also significantly decreased colony size. Antimicrobial peptides such as polymyxin B exacerbated the size difference while human defensin was detrimental to mutant viability. Consistent with the proposed role of MlaA in vesicle biogenesis, the Δ*mlaA* mutant released 1.7-fold more membrane vesicles. Comparative proteomics of cell envelopes and native membrane vesicles derived from Δ*mlaA* and wild type bacteria revealed enrichment of TadA–which recodes proteins through mRNA editing–as well as increased levels of adhesins and virulence factors. MlaA-deficient gonococci significantly outcompeted (up to 16-fold) wild-type bacteria in the murine lower genital tract, suggesting the growth advantage or increased expression of virulence factors afforded by inactivation of *mlaA* is advantageous *in vivo*. Based on these results, we propose *N*. *gonorrhoeae* restricts MlaA levels to modulate cell envelope homeostasis and fine-tune virulence.

## Author summary

The Gram-negative outer membrane is a formidable barrier, primarily because of its asymmetric composition. A layer of lipopolysaccharide is exposed to the external environment and phospholipids are on the internal face of the outer membrane. MlaA is part of a bacterial system that prevents phospholipid accumulation within the lipopolysaccharide layer. If MlaA is removed, membrane asymmetry is disrupted and bacteria become more vulnerable to certain antimicrobials. *Neisseria gonorrhoeae* causes millions of infections worldwide annually. A growing number are resistant to available antibiotics. Improving our understanding of gonococcal pathogenicity and basic biological processes is required to facilitate the discovery of new weapons against gonorrhea. We investigated the role of MlaA in *N*. *gonorrhoeae* and found that when MlaA was absent, bacteria were more sensitive to antibiotics and human defensins. However, the mutant bacteria produced more membrane vesicles–packages of proteins wrapped in membrane material. Mutant vesicles and cell envelopes were enriched in proteins that contribute to disease. These alterations significantly increased mutant fitness during experimental infection of the female mouse genital tract. Our results provide new insights into the processes *N*. *gonorrhoeae* uses to fine-tune its ability to stay fit in the hostile environment of the genital tract.

## Introduction

The Gram-negative cell envelope (CE) plays an important role in bacterial physiology. Not only does it prevent cell lysis through the structure of the peptidoglycan cell wall [1], but it also acts to prevent entry of toxic lipophilic, hydrophilic, and amphipathic molecules. This barrier function is accomplished by the asymmetric outer membrane, which, in contrast to the phospholipid bilayer common to eukaryotic cells, is composed of an outer leaflet of lipopolysaccharide (LPS) or lipooligosaccharide (LOS) and an inner leaflet of phospholipids [2]. Due to the saturated fatty acids found in the hexa-acylated lipid A portion of LPS/LOS, the lipid interior of the LPS/LOS layer is less fluid than that of a phospholipid layer. As a result, the asymmetric bilayer is a more effective barrier than a phospholipid bilayer would be, and is thus less permeable to lipophilic compounds. If the asymmetry of the outer membrane is perturbed, phospholipids diffuse from the inner leaflet to the outer leaflet, which compromises the barrier function of the outer membrane [3].

**Fig 1:**
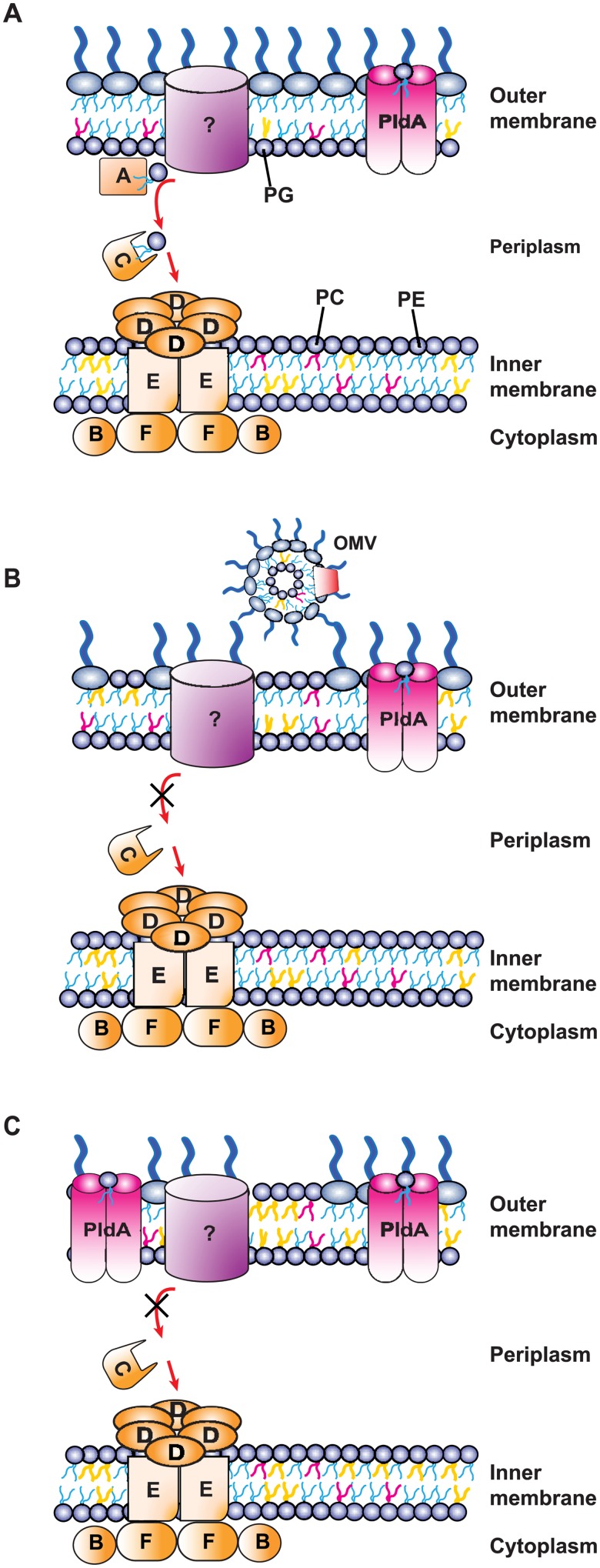
Model of phospholipid homeostasis systems in *N*. *gonorrhoeae*. (A) In WT *N*. *gonorrhoeae*, MlaA, potentially interacting with an unknown partner, participates in retrograde trafficking of phospholipids from the outer leaflet of the outer membrane, through the periplasmic component of the system, MlaC, to the inner membrane MlaBDEF complex. The phospholipase PldA dimerizes to its active form upon detection of mis-localized phospholipids and removes the sn-1 and sn-2 fatty acid side chains. Different phospholipids are represented by lipid tails of different colors (phosphatidylglycerol [PG], yellow; phosphatidylcholine [PC], red; phosphatidylethanolamine [PE], blue). Native *N*. *gonorrhoeae* membrane phospholipid composition can be found in Table 4. (B) When MlaA is removed, phospholipids cannot be transported through the Mla system and invade the outer leaflet of the outer membrane. Increased amounts of membrane vesicles are also produced. (C) When the phospholipase PldA is overexpressed in the absence of MlaA, the PE substrate preference of PldA leads to a membrane phospholipid profile that is skewed toward PG, including in the outer leaflet of the outer membrane. OMV, outer membrane vesicle.

Three lipid asymmetry-maintaining systems, primarily studied in *Escherichia coli*, are the phospholipase A PldA [4], the LPS palmitoyltransferase PagP [5], and the maintenance of lipid asymmetry (Mla) [6] systems. PagP and PldA both remove phospholipids from the outer membrane by destroying the phospholipid. PagP transfers a palmitate residue from the sn-1 position of outer leaflet phospholipids to lipid A to form hepta-acylated LPS, which increases hydrophobic interactions between adjacent LPS molecules [7]. PldA forms an active dimer in the outer membrane in the presence of phospholipids or lyso-phospholipids, then removes the sn-1 and sn-2 fatty acid side chains from the misplaced phospholipids [8]. These fatty acids can act as signal molecules to enhance LPS production, which suggests PldA possesses a secondary function as a sensor for altered membrane homeostasis [9]. In contrast to PagP and PldA, the six-component Mla system, composed of MlaA-F, does not destroy phospholipids in the outer leaflet. Instead, the Mla system is proposed to participate in retrograde transport of phospholipids from the outer leaflet, through the periplasm via MlaC, and back to the inner membrane, where phospholipids are thought to be integrated through the action of the MlaFEDB complex (Fig 1A; [6]). The outer membrane component of this system, MlaA, was recently crystallized from *Klebsiella pneumoniae* and *Serratia marcescens* in complex with the outer membrane β-barrel protein OmpF. The crystal structure revealed that MlaA forms a pore through which phospholipid head groups are able to travel [10]. Loss of MlaA disrupts outer membrane integrity, as demonstrated by increased bacterial sensitivity to a combination of sodium dodecyl sulfate (SDS) and ethylenediaminetetraacetic acid (EDTA), in addition to several antibiotics [6, 10–12]. Single knockouts in any component of the Mla system in *E*. *coli* appear to mirror the defects caused by the lack of MlaA, and double knockouts of *mlaA* with any other Mla component exhibit similar sensitivity to single knockouts [6]. With the exception that iron limitation restricts MlaA transcription *in vitro* and *in vivo* [13], little is known about regulation of the Mla system.

MlaA is present in pathogenic and non-pathogenic Gram-negative bacteria. Its primary function, therefore, appears to be the maintenance of lipid asymmetry. However, infection studies with different pathogenic bacteria suggest that MlaA possesses divergent moonlighting roles. Originally discovered in *Shigella flexneri* and named VacJ for virulence associated, chromosome locus J, this protein contributes to the ability of *S*. *flexneri* to invade adjacent epithelial cells [14, 15]. In *Haemophilus influenzae*, VacJ plays a role in serum resistance [16], and in *Pseudomonas putida* and *Campylobacter jejuni*, MlaA provides protection against oxidative stress [17, 18]. Downregulation of VacJ has also been associated with the increased formation of membrane vesicles in *H*. *influenzae* and *Vibrio cholerae* [13]. MlaA/VacJ knockouts in *S*. *flexneri*, *H*. *parasuis*, and *Salmonella enterica* Typhimurium exhibit virulence defects [11, 14, 15]. In contrast, *P*. *aeruginosa* deficient in VacJ was significantly more virulent [12].

Phospholipid regulation other than the action of PldA has not been studied in the genus *Neisseria*, which includes the human pathogens *N*. *meningitidis* and *N*. *gonorrhoeae*. The *Neisserial* Mla system has not been characterized. *N*. *gonorrhoeae*, the causative agent of gonorrhea, is a worldwide public health threat. The World Health Organization estimates that 78 million new cases are acquired globally every year [19]. Treatment failures with the last effective class of antibiotics have been encountered in several countries, highlighting the necessity of developing new therapeutic interventions [20, 21]. We identified MlaA as a therapeutic candidate for gonorrhea in a high-throughput proteomic examination of the CE and naturally released membrane vesicles (MVs). Deletion of this protein, encoded by the *ngo2121* open reading frame in *N*. *gonorrhoeae* FA1090, resulted in phenotypes that suggested disrupted outer membrane integrity [22]. We subsequently performed a comprehensive phenotypic microarray screen to assess the function of seven proteome-derived gonorrhea vaccine candidates and therapeutic targets, including MlaA, in CE homeostasis. The results revealed an extensive sensitivity phenome in a Δ*mlaA* mutant, including increased susceptibility to compounds that trigger oxidative stress. Importantly, deletion of *mlaA* in the highly antibiotic resistant WHO X strain resulted in similar phenotypes to those observed for a Δ*mlaA* mutant constructed in the FA1090 laboratory strain [23].

In this work, we further characterized the role of MlaA in gonococcal physiology and pathogenesis using different *in silico*, genetic, proteomic, and *in vitro* and *in vivo* assays. The results of our investigations revealed a previously unknown gonococcal virulence pathway and suggest that *N*. *gonorrhoeae* may employ MlaA to modulate its CE and MV protein profile and fine-tune its ability to colonize the host.

## Results

### MlaA conservation

**Fig 2:**
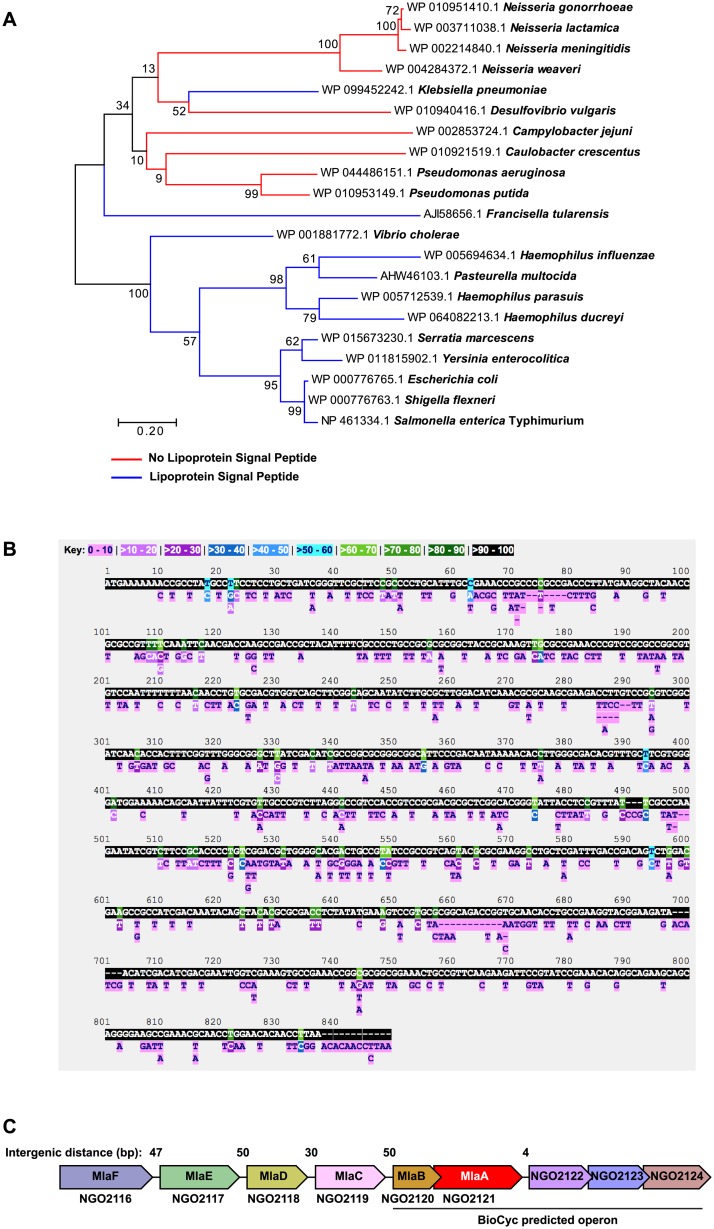
Bioinformatic analysis of MlaA conservation and genome context. (A) A phylogenetic tree of MlaA was constructed in MEGA using amino acid sequences of MlaA/VacJ homologs downloaded from NCBI. The Jones-Taylor-Thornton model was used to generate a pairwise distance matrix. Neighbor-Join and BioNJ algorithms were subsequently applied to the matrix to obtain the initial tree for a heuristic search. 500 bootstrap iterations were performed to test the phylogenies. The highest log-likelihood tree is presented. Homologs without lipoprotein signal peptides are represented by red branches; blue branches represent homologs with lipoprotein signal peptides. (B) The PubMLST *Neisseria* database was used to search for nucleotide polymorphic sites in the *mlaA* (NEIS1933) locus across 44,289 *Neisseria* isolates. (C) Local genome context of *N*. *gonorrhoeae mlaA*. Intergenic distances between open reading frames are noted above the schematic. The operon predicted by biocyc.org is noted below. Schematic and intergenic distances are not to scale.

Despite the importance of MlaA in the Gram-negative cell envelope and its different outcomes on pathogenesis, its conservation across bacterial species has not been addressed. Accordingly, we analyzed the similarity at the amino acid level in a diverse range of Gram-negative bacteria in comparison to *N*. *gonorrhoeae* MlaA. With the exception of other *Neisseria* species, the percent identity was relatively low and ranged from 26.15% to 34.53% (Table 1, S1 File). We previously noted that although *N*. *gonorrhoeae* MlaA is annotated as a predicted lipoprotein, it lacks the universally conserved cysteine residue required for lipidation and membrane anchoring [23, 24]. We were curious whether the lack of a lipoprotein signal peptide is limited to *N*. *gonorrhoeae*; therefore, MlaA homologs from *Neisseria* species and other bacteria in which MlaA/VacJ has been characterized were scrutinized for the presence of a lipoprotein signal peptide. This analysis revealed that homologous proteins in *P*. *aeruginosa*, *P*. *putida*, *C*. *jejuni*, *Caulobacter crescentus*, and *Desulfovibrio vulgaris* did not contain the conserved cysteine residue. Additionally, homologs in the closely-related *N*. *meningitidis*, *N*. *lactamica*, and *N*. *weaveri* did not contain a lipoprotein signal peptide (Table 1). We subsequently carried out a secondary search for MlaA without a predicted lipoprotein signal sequence for the presence of a signal peptidase I (SPaseI) motif and constructed a maximum likelihood phylogenetic tree (Fig 2A). Within the tree, SPaseI-cleaved MlaA homologs clustered separately from those containing a lipoprotein signal peptide (SPaseII-cleaved proteins), with the exception of *K*. *pneumoniae* and *Francisella tularensis*, with the latter protein forming an outgroup within the larger cluster.

**Table 1:** Amino acid identity of MlaA homologs.

Organism with Accession No.	Amino Acid Identity	Predicted Lipoprotein Signal Peptide?	Molecular Mass (kDa)^c^
*Neisseria gonorrhoeae* FA1090 [WP_010951410.1]	100%	No	29.6
*Escherichia coli* [WP_000776765]	28.26%	Yes	28.0
*Neisseria meningitidis* [WP_002214840.1]	95.64%	No	29.5
*Neisseria lactamica* [WP_003711038.1]	96.36%	No	29.5
*Neisseria weaveri* [WP_004284372.1]	63.50%	No	31.5
*Haemophilus influenzae* [WP_005694634.1]	28.38%	Yes	28.1
*Vibrio cholerae* [WP_001881772.1]	28.14%	Yes	28.6
*Shigella flexneri* [WP_000776763.1]	28.26%	Yes	28.0
*Pseudomonas aeruginosa* [WP_044486151.1]	34.53%	No	26.2
*Pseudomonas putida* [WP_010953149.1]	33.93%	No	26.1
*Campylobacter jejuni* [WP_002853724.1]	30.00%	No	26.5
*Salmonella enterica* Typhimurium [NP_461334.1]	28.26%	Yes	28.2
*Pasteurella multocida* [AHW46103.1]	29.52%	Yes	27.6
*Francisella tularensis* [AJI58656.1]	26.15%	Yes	37.5
*Klebsiella pneumoniae* [WP_099452242.1]	34.39%	Yes/No^a^	28.8
*Serratia marcescens* [WP_015673230.1]	29.74%	Yes	28.1
*Haemophilus parasuis* [WP_005712539.1]	27.51%	Yes	28.0
*Yersinia enterocolitica* [WP_011815902.1]	29.18%	Yes	28.1
*Caulobacter crescentus* [WP_010921519.1]	28.86%	No	30.3
*Desulfovibrio vulgaris* [WP_010940416.1]	33.18%	No^b^	32.2
*Haemophilus ducreyi* [WP_064082213.1]	27.63%	Yes	27.7

^a^24 sequences with lipoprotein signal peptide, 3 sequences without any signal peptide

^b^No signal peptide detected.

^c^Molecular mass calculated from unprocessed primary amino acid sequence.

An examination of the level of nucleotide conservation between MlaA homologs in all *Neisseria* performed using the Neisseria Multi Locus Sequence Typing database (locus identifier NEIS1933) indicated that the majority of the *ngo2121* nucleotide sequence represented 90–100% of the alleles, with 461 alleles exhibiting 410 polymorphic sites (Fig 2B). Phylogenetic analyses of amino acid sequences representing MlaA alleles from all *Neisseria* and from *N*. *gonorrhoeae* isolates exclusively showed that MlaA alleles are closely related among *Neisseria* sp. and within *N*. *gonorrhoeae* (S1A and S1B Fig, respectively).

In summary, our examinations of MlaA conservation, both outside and within the *Neisseria* genus, revealed differences that warranted further investigation into the function of this protein in *N*. *gonorrhoeae*.

### Genome context and organization of MlaA

To extend our observations of the differences between MlaA in divergent bacteria, we examined the genomic location of *mlaA* across 7 bacterial species in which MlaA has been investigated. Predictions by biocyc.org suggested that *N*. *gonorrhoeae mlaA* is a member of an operon consisting of *ngo2120* to *ngo2124* (Fig 2C). However, upon closer inspection of the local genome context, *mlaA* appeared to be a part of a polycistronic operon composed of *ngo2116* to *ngo2124*, primarily because of the small intergenic distances and the lack of predicted promoters between each open reading frame. Importantly, NGO2120, NGO2119, NGO2118, NGO2117, and NGO2116 exhibited homology to the other components of the *E*. *coli* Mla system, MlaB, MlaC, MlaD, MlaE, and MlaF, respectively (Fig 2C, Table 1 in S1 Text). The genetic organization surrounding *mlaA* is shared by *N*. *meningitidis* and *N*. *lactamica*. In contrast, *E*. *coli*, *S*. *marcescens*, *S*. *flexneri*, *K*. *pneumoniae*, *P*. *aeruginosa*, and *S*. *enterica* enterica serovar Typhimurium *mlaA* are spatially isolated from the genes encoding the remaining components of the Mla system (S2 Fig). The genomic organization of the Mla system into one operon within the *Neisseria* genomes suggests regulation of Mla complex expression differs between various bacterial species.

### Protein purification and antibody generation

**Fig 3:**
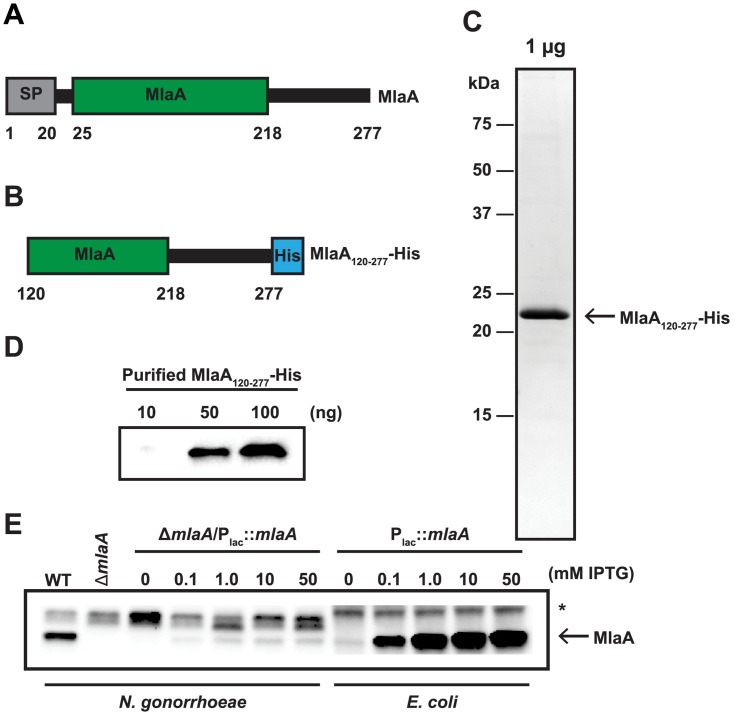
Purification of a truncated recombinant version of MlaA and polyclonal anti-MlaA serum validation. (A) Representative schematic of full length MlaA with annotated MlaA domain. A signal peptide (SP) is noted by a grey rectangle. (B) Schematic of truncated MlaA used for purification with first 119 amino acids removed and a 6 × Histidine tag (His; as a blue rectangle) added to the N-terminus. Schematics are not to scale. (C) Truncated MlaA construct was overexpressed in *E*. *coli* and purified by nickel affinity chromatography in the presence of 1% Triton-X 100 detergent. Detergent was subsequently removed by incubation with Bio-Rad Bio-Beads SM-2 resin. To assess purity, 1 μg of protein was subjected to 1D SDS-PAGE and visualized by staining with Colloidal Coomassie Blue G-250. Open arrow indicates migration band of MlaA_120-277_. Migration of a molecular weight marker (in kDa) is indicated to the left of the gel. (D) Immunoblot evaluation of anti-MlaA antiserum. Indicated amounts of purified MlaA_120-277_ were separated by SDS-PAGE, transferred to a nitrocellulose membrane, and probed with anti-MlaA antiserum. (E) Equivalent OD_600_ units of WT, isogenic Δ*mlaA*, and either Δ*mlaA*/P_lac_::*mlaA* or *E*. *coli* harboring the pGCC4-*ngo2121* complementation plasmid cultured with indicated concentrations of IPTG, were separated by SDS-PAGE, transferred to a nitrocellulose membrane, and probed with anti-MlaA antiserum. Open arrow indicates MlaA. Non-specific cross-reactive band is marked with an asterisk (*). OD_600_, optical density at 600 nm; IPTG, isopropyl β-D-thiogalactopyranoside; SDS-PAGE, sodium dodecyl sulfate-polyacrylamide gel electrophoresis.

To generate molecular tools for our studies, we set out to purify a soluble, recombinant variant of MlaA. A representative schematic of full-length MlaA is presented in Fig 3A. This protein contains a predicted signal peptide from residues 1 to 20, cleaved by SPaseI, and an ABC transporter Mla domain from residues 25 to 218. Although full-length MlaA was predominantly found in the soluble protein fraction, initial purification attempts with a 6× His-tag or a maltose-binding protein purification handle were unsuccessful due to extensive aggregation of MlaA (S3 Fig). To circumvent this challenge, a truncated version of the protein without the first predicted transmembrane helix, MlaA_120-277_, with a C-terminal 6× His-tag was generated (Fig 3B) and purified by affinity chromatography, yielding 99% pure MlaA that migrated at ~22 kDa, consistent with the predicted size of the engineered protein (Fig 3C). Rabbit polyclonal antibodies produced against this protein recognized purified MlaA_120-277_-His (Fig 3D) and a protein band corresponding to the approximate size of native MlaA (29.6 kDa) in whole cell lysates of WT *N*. *gonorrhoeae* but not the Δ*mlaA* mutant (Fig 3E). In all cell lysates, a major cross-reactive protein with highly variable expression that was not associated with MlaA levels was also observed (Fig 3E, marked with an asterisk). BLAST searches of the FA1090 genome with the MlaA_120-277_ amino acid sequence did not reveal any proteins that could be the cross-reactive band. A titration with isopropyl β-D-1-thiogalactopyranoside (IPTG) to examine induction of MlaA in the complemented strain (Δ*mlaA*/P_lac_::*mlaA*) constructed previously [23] revealed that maximal expression of MlaA was achieved with 50 mM IPTG (Fig 3E). However, even with this high level of inducer, expression was not restored to WT levels, indicating that certain phenotypes observed for the *mlaA* null strain may not be entirely complemented, as we previously observed with hypersensitivity to bile salts [23]. A similar IPTG titration performed with an *E*. *coli* strain harboring the pGCC4-*ngo2121* complementation plasmid revealed that MlaA expression was readily induced in a heterologous host (Fig 3E). We independently generated additional complementation strains, one in the FA1090 Δ*mlaA* mutant and four in WHO X lacking MlaA [23]. The non-specific cross-reactive band was not observed in the WHO X strain. WT levels of protein expression were not restored in any of the strains (S4A Fig). These results suggest that MlaA may be under the influence of secondary regulation or requires the presence of additional Mla components for stability in *N*. *gonorrhoeae*.

### Expression of MlaA among gonococcal clinical isolates and within *Neisseria*

**Fig 4:**
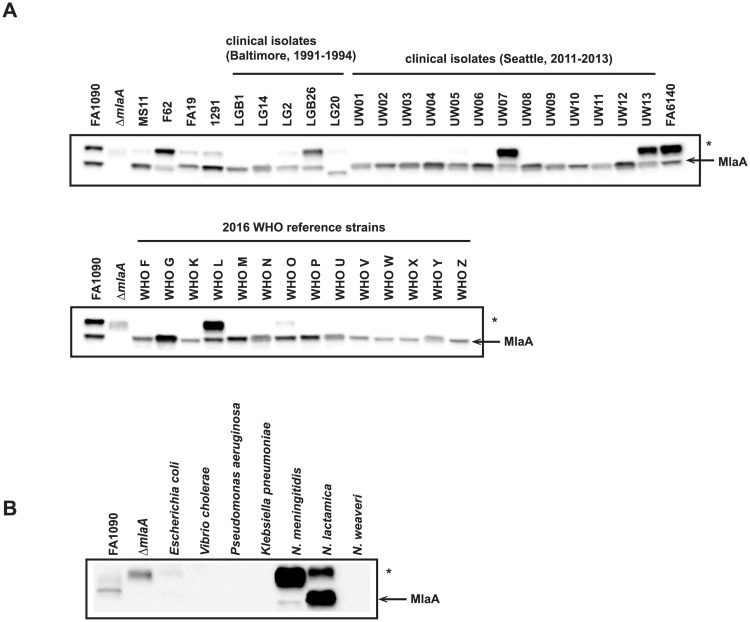
Truncated, recombinant MlaA elicits broadly cross-reactive antisera that recognize MlaA in *Neisseria* species. (A) 37 *N*. *gonorrhoeae* isolates, including common laboratory strains; clinical isolates collected in Baltimore between 1991 and 1994 and Seattle between 2011 and 2013; and the 2016 WHO reference strains were grown on solid media for 20 h at 37 °C in 5% CO_2_. Whole cell lysates were collected and subjected to immunoblotting analysis. (B) Whole cell lysates of different Gram-negative bacteria, including *E*. *coli* BL21(DE3); *V*. *cholerae* N19691; *P*. *aeruginosa* PA01; *K*. *pneumoniae* 6069; *N*. *meningitidis* MC58; the commensal bacterium *N*. *lactamica* NLI83/-01; and the opportunistic pathogen *N*. *weaveri* 1032 were subjected to immunoblot analysis. All lysates were standardized by OD_600_ values, separated in a 4–15% Tris-glycine gel, and probed with polyclonal rabbit antiserum against MlaA. FA1090 and Δ*mlaA* were included in blots as positive controls. Open arrow indicates MlaA. Non-specific cross-reactivity is marked with an asterisk (*). OD_600_, optical density at 600 nm; SDS-PAGE, sodium dodecyl sulfate-polyacrylamide gel electrophoresis.

We examined the expression pattern of MlaA in whole cell lysates collected from geographically and temporally distinct clinical isolates [25, 26]. Immunoblots indicated that MlaA was expressed by all 38 strains at different levels (Fig 4A). Additionally, in the case of isolate LG20, MlaA migrated at a lower molecular weight than FA1090 MlaA. Finally, the non-specific band observed in FA1090 lysates (band marked by asterisk in Fig 4A) was highly abundant only in three of the 18 clinical isolates (LGB26, UW07, and UW13), one WHO reference strain (WHO L), FA6140, and three of the four laboratory strains examined (F62, FA19, and 1291).

We further assessed whether antiserum against FA1090 MlaA could recognize homologous proteins from other *Neisseria* species, as well as more distantly related bacteria. Of the seven species tested, only *N*. *meningitidis* and *N*. *lactamica* homologs were detected (Fig 4B). In summary, these observations provide additional support for the results of our bioinformatic analyses and indicate that MlaA is likely to play a similar role in the CE of clinical isolates collected from different geographical locations, at different points in time, and exhibiting all known antibiotic resistance profiles [25, 26]. Further, the expression of MlaA across a range of diverse clinical isolates supports our use of FA1090 as a type strain to study the effects of the loss of MlaA on gonococcal fitness and pathogenesis.

### Phenotypic characterization of MlaA *in vitro*

**Fig 5:**
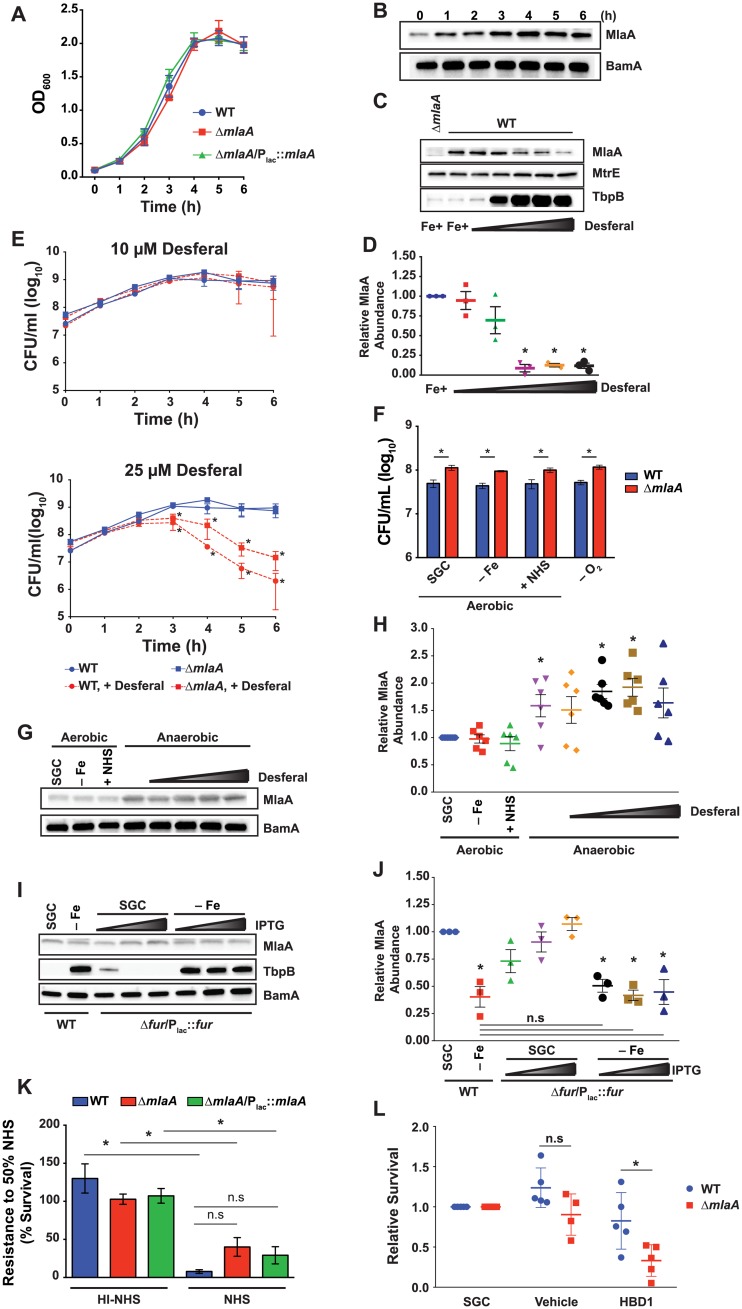
*In vitro* fitness assessments and MlaA expression profiling. (A) WT FA1090, isogenic knockout Δ*mlaA*, and complementation strain Δ*mlaA*/P_lac_::*mlaA* were cultured aerobically in liquid medium. IPTG was added to 0.1 mM in Δ*mlaA*/P_lac_::*mlaA* cultures. Bacterial growth was monitored every hour by OD_600_ measurement. (B) Samples of WT FA1090 were collected at the times indicated. Whole cell lysates were separated by SDS-PAGE and probed with polyclonal rabbit anti-MlaA or anti-BamA (as a loading control) antisera. (C) Whole cell lysates of WT FA1090 were collected after 6 h culture in liquid medium containing desferal at concentrations ranging from 5–25 μM. Samples were probed with antisera against MlaA, MtrE, and TbpB. Δ*mlaA* cultured under standard conditions was included as a reference. (D) Densitometry analyses of MlaA using immunoblots from three independent desferal titration experiments shown as a representative blot in panel (C). (E) WT FA1090 and isogenic Δ*mlaA* were cultured in liquid medium containing either 10 μM (top panel) or 25 μM (bottom panel) desferal for 6 h. At each hour, samples were withdrawn and diluted for CFU/mL enumeration. Both graphs contain growth curves from cultures maintained under standard conditions (blue curves). Statistical significance was assessed by two-way ANOVA using Sidak’s multiple comparisons test. (F) WT FA1090 and isogenic knockout Δ*mlaA* were cultured in liquid medium under standard conditions until OD_600_ had at least doubled (~3 h). Cultures were standardized to an OD_600_ of 0.2 and dilutions were spotted onto GCB. Plates were prepared as normal (SGC); under iron deprivation (-Fe); supplemented with 7.5% normal human serum (+NHS); or supplemented with 1.2 mM NaNO_2_ and cultured anaerobically (-O_2_). Strains were maintained at 37 °C in 5% CO_2_ for approximately 22 h or at 37 °C anaerobically for 48 h and CFU were enumerated. (G) WT bacteria cultured as in (F), with the addition of a desferal titration from 0–25 μM under anaerobiosis, were collected from plates, separated by SDS-PAGE, and probed with polyclonal rabbit anti-MlaA antiserum or anti-BamA antiserum as a loading control. (H) Densitometry analyses of MlaA abundance under each host relevant condition presented in (G). Densitometry was performed twice on each of three independent experiments. (I) WT FA1090 and conditional knockout Δ*fur*/P_lac_::*fur* were cultured in liquid medium in the absence (SGC) or presence of 25 μM desferal (-Fe). Fur expression was induced by the addition of 10, 50, or 100 μM IPTG. Samples were collected after 6 h of growth and probed with indicated antisera. (J) Densitometry analyses of MlaA abundance in immunoblots from three independent Fur induction experiments with and without iron starvation. (K) WT FA1090, isogenic knockout Δ*mlaA*, and Δ*mlaA*/P_lac_::*mlaA* were cultured aerobically in liquid medium until culture density had doubled (~3 h). Cultures were diluted to an OD_600_ of 0.05 in sterile PBS and diluted 1000-fold in EMEM. Suspensions were combined with an equal volume of EMEM, NHS, or heat-inactivated NHS and incubated for 1 h in 5% CO_2_ at 37 °C. Bacteria from each well were spotted onto GCB plates. CFUs were scored after 20–22 h incubation in 5% CO_2_ at 37 °C (*p* value between WT and Δ*mlaA* exposed to active NHS, 0.07). (L) Rapidly growing cultures of WT FA1090 and isogenic knockout Δ*mlaA* were diluted to 10^5^ CFU/mL and cultured for 3 h in the presence of liquid medium (SGC), 0.01% acetic acid (vehicle), or 10 μM human defensin (HBD1). Bacteria were serially diluted and spotted onto GCB plates. CFUs were scored after 20–22 h incubation in 5% CO_2_ at 37 °C and survival was calculated relative to SGC. *n*≥3; mean ± SEM presented for all experiments; panels D, H, J, and L present values from each replicate; *p < 0.05; OD_600_, optical density at 600 nm; SGC, standard growth conditions; IPTG, isopropyl β-D-thiogalactopyranoside; SDS-PAGE, sodium dodecyl sulfate-polyacrylamide gel electrophoresis; CFU, colony forming unit; GCB, gonococcal base medium; GCBL, gonococcal base liquid medium; PBS, phosphate buffered saline; EMEM, Eagle’s minimal essential medium.

To gain further insights into the impact of MlaA on gonococcal physiology, we first examined the growth kinetics of the WT and Δ*mlaA* mutant under standard growth conditions in liquid medium. Neither the Δ*mlaA* mutant nor the complemented strain displayed a difference in growth compared to WT (Fig 5A), consistent with our previous observations using solid medium and chemically defined Graver-Wade liquid medium [22, 23]. An assessment of MlaA abundance in WT whole cell lysates from liquid cultures over time revealed that expression of MlaA was lower during the lag phase, slightly increased during logarithmic growth, reached maximum expression at approximately mid-logarithmic phase, and was maintained at similar levels until stationary phase (Fig 5B), at which point the experiment was terminated to avoid the effects of autolysis [27].

### *N*. *gonorrhoeae* restricts the MlaA cellular pool during iron deprivation

Iron is a well-characterized regulator of gonococcal gene expression. Numerous genes are iron-repressed and are thus expressed in body sites where iron is limited [28, 29]. Therefore, we assessed the expression of *N*. *gonorrhoeae* MlaA and its effect on bacterial viability under iron deprivation. Titration with the iron chelator desferal (up to 25 μM) revealed that MlaA production decreased under increasing iron limitation, in contrast to TbpB, a well-characterized protein induced during iron starvation ([30]; Fig 5C). Antiserum against MtrE, a component of the MtrCDE efflux pump that is not influenced by iron [25, 31], was used as a loading control and showed that MtrE expression was unaffected under any of the desferal concentrations tested. Densitometry analysis of MlaA abundance indicated a statistically significant decrease in the MlaA cellular pool under exposure to 15 μM desferal and higher (Fig 5D).

To examine whether the lack of MlaA affects bacterial viability under various iron concentrations, we monitored the growth of WT and Δ*mlaA* bacteria over time by enumeration of colony forming units (CFU/mL). Both strains were equivalently viable when exposed to moderate iron starvation (10 μM desferal; Fig 5E, top panel). In contrast, exposure to high iron starvation (25 μM of the iron chelator) resulted in a significant decrease in bacterial viability beginning at 3 h and continuing to the experimental endpoint for both strains, with the Δ*mlaA* mutant exhibiting a slight, non-statistically significant growth advantage over the WT (Fig 5E, bottom panel).

Together, these results show that depletion of iron negatively regulates the MlaA cellular pool and suggest that decreased levels of MlaA are favored by the bacteria under iron starvation encountered in the host.

### MlaA affects bacterial viability under other *in vitro* conditions relevant to infection

*N*. *gonorrhoeae* is also exposed to conditions other than iron limitation during infection of different niches within the human host. Gonococci proliferate in microaerobic or anaerobic conditions within the female reproductive tract and may also be exposed to serum within inflammatory exudates and during disseminated infections [32]. The recovery of viable Δ*mlaA* mutant bacteria was statistically significantly higher (~2-fold) than that of the WT for all tested conditions relevant to infection of the host using solid medium with 5 μM desferal (- Fe), 7.5% normal human serum (NHS), or with nitrite under anaerobic conditions (- O_2_; Fig 5F). As expected from our investigations into iron starvation in liquid medium, immunoblotting analysis indicated no difference in MlaA expression under aerobic exposure to 5 μM desferal. The presence of serum also had no effect on MlaA levels. In contrast, MlaA was elevated under anaerobiosis in comparison to standard growth (Fig 5G). To examine the interplay between iron starvation and anoxia on MlaA expression, immunoblotting was also performed on bacteria collected from anaerobic conditions with increasing concentrations of desferal. Densitometry analysis indicated a significant difference in the abundance of MlaA during anoxia, as well as during anaerobic exposure to 10 or 20 μM desferal, although MlaA expression was highly variable under each anaerobic condition tested (Fig 5H). The integral membrane component of the β-barrel assembly machinery (BAM) complex, BamA [25, 33], was used as a loading control (Fig 5G), and its expression was not altered under any of the conditions examined [25].

### Iron starvation diminishes the MlaA cellular pool independent of Fur levels

In *N*. *gonorrhoeae*, the ferric uptake regulator (Fur) protein governs expression of iron homeostasis genes in response to the intracellular pools of this important metal. To dissect the mechanism of MlaA repression during iron deprivation, we employed a conditional Fur knockout, Δ*fur*/P_lac_::*fur*, as Fur is essential in *N*. *gonorrhoeae* (S5A Fig, [34]). Initial viability assessment indicated that Δ*fur*/P_lac_::*fur* proliferated identically to WT bacteria in liquid medium supplemented with IPTG, both in the presence and absence of desferal (S5B Fig). Immunoblotting (Fig 5I) coupled with densitometry revealed a statistically significant decrease in MlaA levels under iron starvation, which was not restored with low (10 μM IPTG) or high (100 μM IPTG) Fur induction (Fig 5J). A non-significant reduction in MlaA expression compared to standard conditions was observed with the lowest Fur level examined during iron repletion, and MlaA abundance trended upward as IPTG was added (Fig 5J). We also examined TbpB expression as a Fur-regulated control [35]. Our analysis indicated that low Fur expression during iron repletion was insufficient to repress TbpB to the levels observed for the WT under standard conditions (Fig 5I and S5C Fig). Upon iron depletion, TbpB was derepressed in the Δ*fur*/P_lac_::*fur* strain, regardless of Fur induction (Fig 5I), reflecting the inability of the repressor to dimerize and bind to its DNA target in the absence of iron [36]. BamA was used as a loading control and exhibited no alterations under any condition examined. Our quantitative immunoblotting assessments suggest Fur may exert a slight positive effect on MlaA expression under iron replete conditions, but that iron starvation overrules this influence. It is possible that MlaA is regulated by several factors–similar to TbpB, which is controlled by a long non-coding RNA and the MisR response regulator [28, 36].

### *N*. *gonorrhoeae* MlaA does not contribute to serum resistance

Next, we sought to examine the sensitivity of WT, Δ*mlaA*, or Δ*mlaA*/P_lac_::*mlaA* bacteria to NHS in an assay using liquid medium [37], as VacJ has been described to contribute to serum resistance in *H*. *influenzae* [16]. Enumeration of CFUs after exposing gonococci to 50% NHS for 1 h revealed a significant decrease in viability for all strains compared to the same concentration of heat-inactivated NHS. No significant difference in survival, however, was observed between the WT, Δ*mlaA*, or Δ*mlaA*/P_lac_::*mlaA* strains during exposure to active serum (Fig 5K).

### Bacteria lacking MlaA are more sensitive to human defensins

To extend our examination into the possible physiological contribution of MlaA during infection of the host, we exposed WT and Δ*mlaA* bacteria to human β-defensin 1 (HBD1). Defensins are cationic antimicrobial peptide components of the innate immune system which interfere with membrane integrity to exert their antibacterial effect [38]. Lack of MlaA was associated with a significant 2.5-fold reduction in viability compared to WT after HBD1 treatment (Fig 5L). *N*. *gonorrhoeae* is naturally resistant to HBD1 [39], which is constitutively expressed by epithelial cells [38]. The Δ*mlaA* mutant’s diminished viability upon HBD1 exposure suggests that loss of MlaA results in a cell envelope defect.

### Outer membrane integrity is altered in a *mlaA* null mutant and can be partly rescued by PldA overproduction

**Fig 6:**
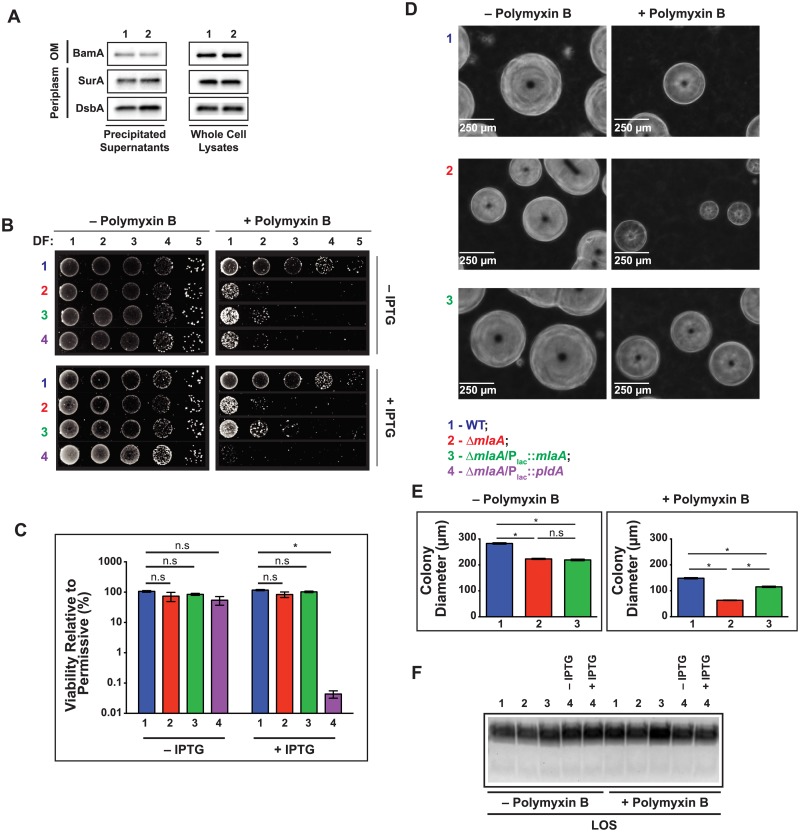
Loss of MlaA results in a reduction in gonococcal colony size that is exacerbated in the presence of the antimicrobial peptide polymyxin B. (A) Supernatants from mid-logarithmic cultures of WT and Δ*mlaA* bacteria were separated by low-speed centrifugation and filtration, treated with DNAseI, and precipitated with a pyrogallol red-molybdate-methanol procedure. Precipitated supernatants and whole cell lysates were standardized by the OD_600_ of the source culture, separated by SDS-PAGE, and probed with indicated antisera. (B) WT FA1090, isogenic knockout Δ*mlaA*, complementation strain Δ*mlaA*/P_lac_::*mlaA*, and PldA overexpression strain Δ*mlaA*/P_lac_::*pldA* were cultured aerobically in liquid medium for 3 h, back diluted to an OD_600_ of 0.1, cultured 2 h longer, serially diluted, and spotted onto GCB without (left column) or with (right column) polymyxin B (800 U/mL) and either without (top row) or with (bottom row) 0.5 mM IPTG. Dilution spots from each condition were imaged with a Bio-Rad ImageDoc system. (C) CFUs for permissive and restrictive conditions with or without IPTG were counted and relative viability was calculated. Experiment was performed on three separate occasions (mean ± SEM on graph; *p < 0.05), and typical plate images are presented. (D) Representative micrographs from 10^−4^ dilution taken with a Zeiss AxioObserver.D1 microscope at 10× magnification 0.25 Phase Contrast 1 of WT (Row 1), Δ*mlaA* (Row 2), and Δ*mlaA*/P_lac_::*mlaA* (Row 3). MlaA expression was induced by inclusion of 0.1 mM IPTG in the solid medium for the complementation strain. (E) Images of 10^−4^ dilution were also taken at 2.5× magnification 0.06 Phase Contrast 1 and colony diameters were measured with ImageJ software. Colonies were measured for each of two independent experiments for the − polymyxin B condition (WT, *n* = 548; Δ*mlaA*, *n* = 755; Δ*mlaA*/P_lac_::*mlaA*, *n* = 664) and for the + polymyxin B condition (WT, *n* = 836; Δ*mlaA*, *n* = 1197; Δ*mlaA*/P_lac_::*mlaA*, *n* = 1121; mean ± SEM on graphs; *p < 0.05). (F) Rapidly growing liquid cultures of WT, Δ*mlaA*, Δ*mlaA*/P_lac_::*mlaA*, and Δ*mlaA*/P_lac_::*pldA* incubated in the presence or absence of polymyxin B were lysed and treated with proteinase K to isolate LOS. Subsequently, LOS was separated by SDS-PAGE and visualized by silver staining. IPTG was added to Δ*mlaA*/P_lac_::*mlaA* and Δ*mlaA*/P_lac_::*pldA* cultures to 0.5 mM as indicated. DF, dilution factor; OD_600_, optical density at 600 nm; IPTG, isopropyl β-D-thiogalactopyranoside; LOS, lipooligosaccharide; SDS-PAGE, sodium dodecyl sulfate–polyacrylamide gel electrophoresis.

Our observation that the Δ*mlaA* mutant was more susceptible to the membrane perturbing activity of HBD1 is consistent with our previous studies, in which we reported increased sensitivity of Δ*mlaA* mutants in the FA1090 and WHO X genetic backgrounds to polymyxin B on solid medium [22, 23]. Gonococci lacking MlaA were also more susceptible to several antibiotics during growth in chemically defined liquid medium, a sensitivity phenome that suggested an outer membrane defect, rather than general loss of membrane integrity [23]. To further determine how the altered outer membrane integrity in the Δ*mlaA* mutant translates to antibiotic tolerance, we applied an alternative approach and examined bacterial susceptibility to ten antibiotics with different mechanisms of action using Etest strips (Table 2). As expected from our previous studies, loss of MlaA resulted in a 2-fold decrease in the minimal inhibitory concentration (MIC) of polymyxin B (Δ*mlaA* mutant, MIC 32 μg/mL; WT bacteria, MIC 64 μg/mL), ampicillin (0.064 μg/mL, versus 0.125 μg/mL against the WT), and vancomycin (4 μg/mL, versus 8 μg/mL against the WT). All three antimicrobial compounds act by interfering with the barrier function of the outer membrane and the cell wall [40], and vancomycin was selected specifically to serve as a marker for outer membrane permeability, as an intact Gram-negative outer membrane excludes this antibiotic [41]. No differences in the MICs of other antibiotics with different mechanisms of action were observed. Consistent with results in the FA1090 background, deletion of *mlaA* from the WHO X genome resulted in 16- and 2-fold lower polymyxin B and vancomycin MICs, respectively (Table 2). Additionally, the clinically relevant antibiotics ceftriaxone and cefixime were not more effective against the WHO X Δ*mlaA* mutant, which provided further evidence that the permeability defect solely affects the outer membrane, as observed for the FA1090 *mlaA* null strain. On the other hand, the loss of MlaA does not interfere with cell envelope integrity to the extent that periplasmic proteins are released to the extracellular milieu, as no increase in the abundance of DsbA or SurA was observed in Δ*mlaA*–derived supernatants (Fig 6A). Additionally, neither DsbA nor SurA was upregulated in whole cell lysates of the Δ*mlaA* mutant, suggesting that absence of MlaA does not interfere with proper outer membrane protein folding [42–44].

**Table 2:** Etest assessments of cell envelope integrity.

	FA1090 background			WHO X background	
	WT^a^	Δ*mlaA*^a^	Δ*mlaA*/P_lac_::*pldA*^a^	WT^a^	Δ*mlaA*^a^
**Polymyxin B**	64	32	16	128	8
**Vancomycin**	8	4	4	16	8
**Ceftriaxone**^b^	N/D	N/D	N/D	1.0^d^	1.0
**Cefixime**^b^	N/D	N/D	N/D	2.0^d^	2.0
**Azithromycin**	0.064	0.064	1.0^c^	N/D	N/D
**Cefotaxime**	0.004	0.004	0.004	N/D	N/D
**Ampicillin**	0.125	0.064	0.125	N/D	N/D
**Tetracycline**	0.125	0.125	0.125	N/D	N/D
**Benzylpenicillin**	0.064	0.064	0.064	N/D	N/D
**Gentamicin**	4	4	4	N/D	N/D
**Tobramycin**	8	8	8	N/D	N/D
**Ceftazidime**	0.032	0.032	0.032	N/D	N/D

^a^MIC values are presented in μg/mL.

^b^Preliminary testing indicated FA1090 was too sensitive to this antibiotic for the MIC to be evaluated with Etests.

^c^Vector for PldA overexpression encodes erythromycin resistance, which also provides resistance against azithromycin.

^d^Prior MIC determination performed on solid medium supplemented with hemoglobin and IsoVitalex [26]. N/D, not determined.

Overexpression of the phospholipase PldA rescued SDS/EDTA sensitivity in *E*. *coli* Δ*mlaC* and in Δ*mlaA*/Δ*mlaC* knockouts, presumably by removal of phospholipids accumulated at the cell surface [6]. We therefore determined whether PldA overproduction would rescue the antibiotic sensitivity phenotypes observed for the *N*. *gonorrhoeae* Δ*mlaA* mutant. While WT resistance to ampicillin was restored in the Δ*mlaA*/P_lac_::*pldA* strain, the vancomycin MIC was the same as the Δ*mlaA* strain. However, when PldA was overexpressed, the strain’s polymyxin B sensitivity increased two- and four-fold in comparison to the Δ*mlaA* mutant and the WT strain, respectively (Table 2). Finally, follow-up agar dilution MIC assessment confirmed the fold decrease in MIC for each strain for polymyxin B and vancomycin but not for ampicillin. Complementation with MlaA was sufficient to restore resistance to polymyxin B but not to vancomycin (Table 2 in S1 Text). In all cases, the MICs were higher by agar dilution than by Etest, an effect noted previously [45].

### Loss of MlaA results in reduction of gonococcal colony size

Our antibiotic susceptibility testing indicated Δ*mlaA* exhibited an outer membrane defect that was exacerbated upon overproduction of PldA in the presence of polymyxin B. To examine these effects more closely, viability was assessed for WT, Δ*mlaA*, Δ*mlaA*/P_lac_::*mlaA*, and Δ*mlaA*/P_lac_::*pldA* grown on solid medium supplemented with 800 U/mL (~84 μg/mL) polymyxin B and either 0 or 0.5 mM IPTG for the expression of MlaA or PldA. Visual inspection revealed an apparent decrease in Δ*mlaA* viability from the -5 to the -3 dilution during exposure to polymyxin B (Fig 6B, top panels). Upon examination with a stereo-microscope, colonies of the Δ*mlaA* mutant were noticeably smaller than those of the WT, although colony counting revealed no significant difference in CFUs compared to the absence of polymyxin B for the Δ*mlaA* mutant (106.2% ± 6.8 for WT compared to 73.6% ± 24.5 for Δ*mlaA*). In the absence of PldA induction, the viability of the Δ*mlaA*/P_lac_::*pldA* strain was not significantly different from that of the WT when exposed to polymyxin B (54.2% ± 17.42 for Δ*mlaA*/P_lac_::*pldA*). However, upon *pldA* overexpression in the Δ*mlaA* background, survival was significantly lower than WT bacteria (117.2 ± 5.7 for WT; 0.04% ± 0.01 for Δ*mlaA*/P_lac_::*pldA*), dropping by 99.96% compared to cells cultured in the absence of polymyxin B (Fig 6C).

Measurement of colony diameters (Fig 6D, left column) revealed that lack of MlaA resulted in significantly decreased colony size, even in the absence of the antimicrobial peptide (282.7 ± 2.66 μm for WT, 223.2 ± 2.19 μm for Δ*mlaA*). This phenotype was not reversed in the Δ*mlaA* mutant by expression of MlaA from a heterologous location (219.1 ± 2.91 μm; Fig 6E, left panel). The difference in colony size between WT and Δ*mlaA* was aggravated in the presence of polymyxin B (148.6 ± 2.35 μm for WT, 63.28 ± 1.02 μm for Δ*mlaA*; Fig 6D, right column), and was partially restored in the Δ*mlaA*/P_lac_::*mlaA* strain (115.4 ± 2.45 μm; Fig 6E, right panel). Analysis of LOS isolated from WT, Δ*mlaA*, Δ*mlaA*/P_lac_::*mlaA*, and with PldA at either native levels or overproduced in Δ*mlaA*/P_lac_::*pldA* bacteria in the presence or absence of polymyxin B revealed that neither loss of MlaA nor overproduction of PldA in the Δ*mlaA* background resulted in alterations to LOS abundance or migration (Fig 6F). Likewise, the presence of the antimicrobial peptide had no effect on LOS in any of the strains tested. Thus, the polymyxin B phenotype was not due to LOS defects.

Our experiments provided the first evidence that in *N*. *gonorrhoeae*, absence of MlaA affects outer membrane permeability to compounds acting against the cell wall, which can be partly rescued by the action of PldA, and alters colony morphology. Further, the relatively low levels of MlaA present in the Δ*mlaA*/P_lac_::*mlaA* strain partially alleviated the polymyxin B sensitivity phenotype resulting from this defect (Fig 3E). We conclude that MlaA is more important to the bacteria during exposure to cell envelope stress conditions than under standard laboratory growth.

### Gonococcal cell morphology is unaltered in the absence of MlaA

**Fig 7:**
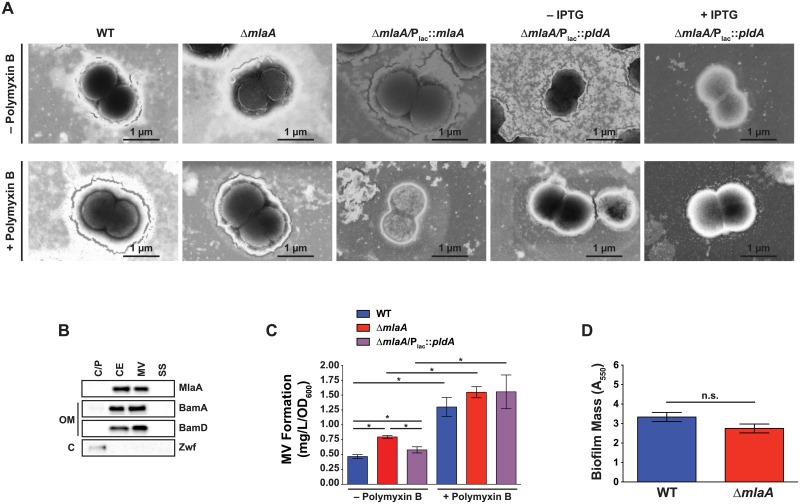
Investigations of MlaA effects on cell structure, membrane vesicles, and biofilm formation. (A) Strains as indicated were cultured in liquid medium without (top row) or with (bottom row) polymyxin B until approximately mid-logarithmic growth. Δ*mlaA*/P_lac_::*pldA* was cultured in the presence or absence of 0.5 mM IPTG, as indicated. Bacteria were subsequently washed twice with PBS, spotted onto 300 mesh copper grids, negatively stained with phosphotungstic acid, and imaged using scanning electron microscopy. (B) Cytoplasmic/periplasmic (C/P), cell envelope (CE), membrane vesicle (MV), and soluble supernatant (SS) subcellular fractions of WT FA1090 collected under standard aerobic conditions were prepared and normalized based on protein concentration, separated by SDS-PAGE, and probed with indicated antisera. (C) MVs collected by ultracentrifugation of culture supernatants derived from strains indicated were quantified by protein concentration (*n* = 6 for WT and Δ*mlaA* for − polymyxin B condition; *n* = 3 for all others; mean ± SEM). (D) WT FA1090 and Δ*mlaA* bacteria were suspended to an OD_550_ of 1.5 in GCBL, added to 96 well microtiter plates, and cultured without shaking in 5% CO_2_ at 37 °C for 24 h. Planktonic bacteria were removed and biofilms were washed with PBS. Biofilms were allowed to dry at room temperature, then stained in 0.1% crystal violet in 2% ethanol. After staining, wells were washed with PBS and dried. Biofilms were dissolved in 30% acetic acid and quantified by A_550_ measurement. Biofilm experiments were performed 12 times, each with 3 or 4 technical replicates, for a total of 46 datapoints. Mean ± SEM is presented. IPTG, isopropyl β-D-thiogalactopyranoside; C/P, cytoplasmic/periplasmic; C, cytoplasm; CE, cell envelope; OM, outer membrane; MV, membrane vesicle; SS soluble supernatant; SDS-PAGE, sodium dodecyl sulfate-polyacrylamide gel electrophoresis; OD_550_, optical density at 550 nm; A_550_, absorbance at 550 nm, GCBL, gonococcal base liquid medium.

To determine whether the loss of MlaA either alone or combined with PldA expression would alter cell morphology and thus result in increased sensitivity to polymyxin B, we employed transmission electron microscopy. Cells from the Δ*mlaA* mutant typically appeared identical to WT cells, although the mutant occasionally exhibited ruffled membranes (Fig 7A, top row). Additionally, the Δ*mlaA*/P_lac_::*pldA* strain was indistinguishable from WT, either in the presence or absence of PldA overexpression (Fig 7A, top row). To determine whether any of the strains exhibited morphological differences in the presence of polymyxin B, we also imaged diplococci collected from cultures supplemented with the antimicrobial peptide. The presence of polymyxin B did not alter the overall morphology of any of the strains examined (Fig 7A, bottom row). Finally, we occasionally observed numerous blebs on cell surfaces, as shown for WT bacteria cultured without polymyxin B and Δ*mlaA* bacteria collected from both conditions (Fig 7A).

### MlaA localizes to the cell envelope and naturally released membrane vesicles

We identified MlaA within CE and MV fractions isolated from four different *N*. *gonorrhoeae* strains in proteomic investigations using isobaric Tagging for Relative and Absolute Quantification coupled with Mass Spectrometry [22]. To validate these results and to determine to which cellular compartment(s) *N*. *gonorrhoeae* MlaA associates, we performed an immunoblotting analysis of subcellular fractions using anti-MlaA antiserum and control antisera against the periplasmic-facing lipoprotein member of the BAM complex, BamD [46]; BamA [25, 33]; and the cytoplasmic enzyme Zwf [47–49]. This analysis revealed that none of the proteins were detected in the supernatant (SS). MlaA exclusively localized to the CE and MV fractions, similar to BamD. BamA was detected primarily in the CE and MV fractions, but was also found in small amounts in the cytoplasmic/periplasmic fraction, which reflects the presence of five periplasmic polypeptide transport associated domains [25, 33]. Finally, as expected [49], Zwf was detected solely in the cytoplasmic/periplasmic fraction (Fig 7B).

### Cell envelope and membrane vesicle composition is altered in the Δ*mlaA* mutant

The presence of membrane blebs on the surface of Δ*mlaA* cells both under permissive and stress conditions (Fig 7A), combined with the proposed role of MlaA in vesicle biogenesis [13], prompted us to assess whether this mechanism was conserved in *N*. *gonorrhoeae*. We examined vesicle formation by WT, Δ*mlaA*, and Δ*mlaA*/P_lac_::*pldA* bacteria during standard growth in liquid medium, as well as under polymyxin B exposure. Indeed, quantitation of MVs revealed that the Δ*mlaA* mutant produced significantly more vesicles than WT under standard conditions (0.80 ± 0.02 mg L^-1^OD_600_^-1^ compared to 0.47 ± 0.03 mg L^-1^OD_600_^-1^ for WT, Fig 7C). Overexpression of PldA partially decreased MV production, although the Δ*mlaA*/P_lac_::*pldA* strain still released significantly more MVs than WT bacteria (0.58 ± 0.03 mg L^-1^OD_600_^-1^, Fig 7C). However, in the presence of polymyxin B, all three strains produced significantly more MVs than under non-stress conditions (2.8-, 1.9-, and 2.7-fold increases for WT, Δ*mlaA*, and Δ*mlaA*/P_lac_::*pldA*, respectively). No significant differences were observed in MV secretion between strains during polymyxin B exposure (Fig 7C). The hypervesiculation phenotype of the Δ*mlaA* mutant did not translate into increased biofilm formation (Fig 7D, S6 Fig) despite the fact that gonococcal biofilms are primarily membranous material generated by the release of MVs [50].

### MlaA reduces gonococcal fitness in the female mouse lower genital tract

**Fig 8:**
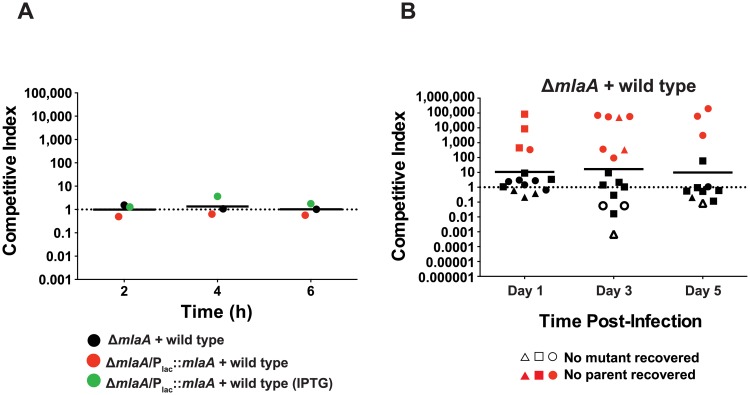
MlaA influences gonococcal fitness *in vivo*. (A) *In vitro* competition assays were performed by combining WT FA1090 bacteria with approximately equal numbers of Δ*mlaA* or Δ*mlaA*/P_lac_::*mlaA* (~10^6^ CFU total bacteria). Competitions were carried out in liquid medium, and output CFUs were assessed at 2, 4, and 6 h post-inoculation. Competitions with the complemented strain were performed in liquid medium both with and without IPTG. (B) Female BALB/c mice were inoculated intravaginally with approximately equal numbers of CFUs of WT and Δ*mlaA* bacteria (~10^6^ CFU total *N*. *gonorrhoeae*; 7 mice per group). Vaginal swabs were taken on days 1, 3, and 5 post-infection and were cultured for CFU/mL enumeration on solid media containing streptomycin (total bacteria) or media containing streptomycin and kanamycin (Δ*mlaA* bacteria). Experiments were repeated three times and results are expressed as the geometric mean of the competitive index (CI): [mutant CFU (output) / WT CFU (output)] / [mutant CFU (input) / WT CFU (input)]. A CI > 1 indicates that the mutant was more fit during the competition. 1 CFU was assigned for any strain not recovered from an infected mouse. IPTG, isopropyl β-D-thiogalactopyranoside; CFU, colony forming unit.

The increased blebbing, sensitivity to antimicrobial peptides, and the differential expression pattern of MlaA during iron deprivation and anaerobiosis led us to test whether the lack of MlaA impacts *N*. *gonorrhoeae* survival during infection. We chose to establish a competitive index (CI) as it provides a relative measure of bacterial fitness in comparison to a reference strain (in this case, WT bacteria) that takes the starting inoculum into account (see Materials and methods for calculation). A CI equal to 1 indicates that both strains are comparably fit whereas CI values greater or lower than 1 show that the mutant is able to outcompete the WT strain or displays decreased fitness, respectively. We first established that MlaA does not affect bacterial fitness using *in vitro* competitive experiments (Fig 8A) then used the female mouse model of gonococcal lower genital tract infection as a measure of *in vivo* fitness [51, 52]. In three biological replicates, each with at least seven mice, we observed a 9.8- to 16.9-fold increase in the competitive index for the Δ*mlaA* strain. Additionally, only mutant bacteria were isolated from several mice at each time point (Fig 8B). Expectedly, the same phenotype was observed in the Δ*mlaA*/P_lac_::*mlaA* strain (S7 Fig) due to the inability to restore MlaA expression to WT levels in this complemented strain (Fig 3E).

### Quantitative proteomic profiling reveals significant differences in the cell envelope and membrane vesicles upon loss of MlaA

**Fig 9:**
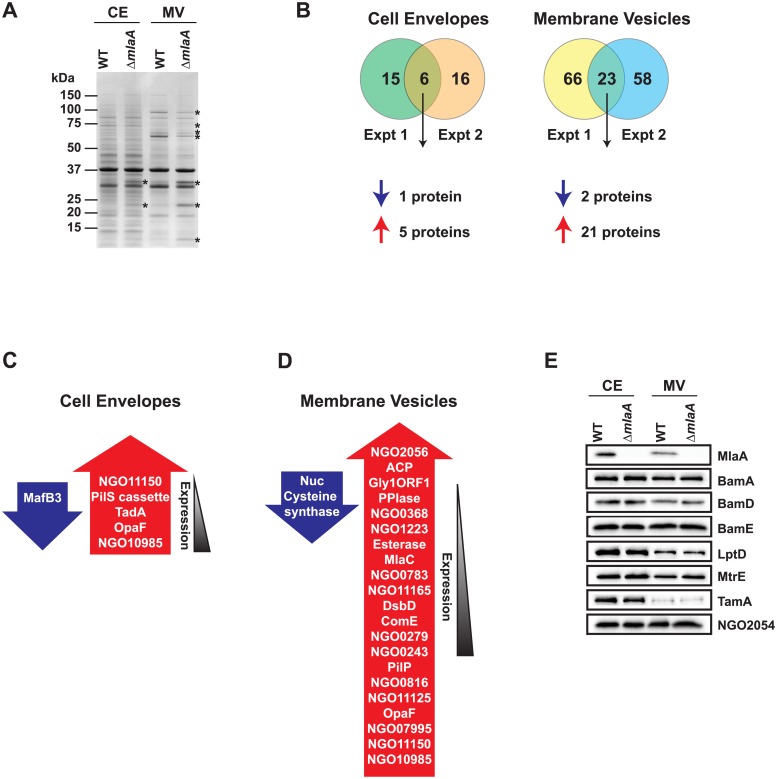
Proteomic investigations of MlaA influence on cell envelope and membrane vesicles. (A) CE and MV fractions isolated from WT FA1090 and isogenic knockout Δ*mlaA* were normalized based on protein concentration, separated by SDS-PAGE, and proteins were visualized by coomassie staining. The migration of a molecular weight marker is shown on the left in kDa. Proteins that appeared differentially abundant in the Δ*mlaA* mutant by visual inspection are labeled with an asterisk. (B) Trypsinized CE and MV proteins from WT and Δ*mlaA* were labeled with TMT6plex isobaric mass tags, fractionated by strong cation exchange and reverse phase chromatography, and subjected to peptide identification by tandem mass spectrometry. The number of differentially abundant proteins in the Δ*mlaA* CE or MV protein profiles is noted in the Venn diagrams. (C, D) Lists of differentially abundant proteins in the CE (C) or MVs (D) of the Δ*mlaA* mutant. Proteins in blue arrows are downregulated in the mutant, while those listed in red arrows are upregulated in the mutant. Proteins are arranged by the magnitude of the mutant:WT ratio. (E) Validation of quantitative proteomics results. CE and MV fractions from WT FA1090 and Δ*mlaA* were normalized by protein concentration, separated by SDS-PAGE, and probed with antisera against indicated proteins. SDS-PAGE, sodium dodecyl sulfate-polyacrylamide gel electrophoresis; TMT, tandem mass tag.

We hypothesized that the enhanced fitness of the Δ*mlaA* strain may be due to increased MV formation, as well as specific changes to CE and MV subproteomes (Fig 9A). To quantitatively examine these alterations, we utilized Tandem Mass Tag (TMT) 6plex isobaric mass tags to label trypsinized proteins from CE and MV fractions collected from WT and Δ*mlaA* bacteria on two separate occasions. Proteins were considered differentially expressed in the mutant if the ratio of Δ*mlaA* protein abundance to WT protein abundance was greater than 1.5-fold (increased in Δ*mlaA*) or less than 0.67-fold (decreased in Δ*mlaA*). Differentially expressed proteins in CE and MV fractions are listed in Table 3 and Fig 9C and 9D, and proteomic data are presented in S2 and S3 Files, respectively. All MS data are also available via ProteomeXchange with identifier PXD008673.

**Table 3:** Differentially expressed proteins in Δ*mlaA* cell envelopes and membrane vesicles identified in two biological replicate experiments.

Accession	Protein Name	Locus	Molecular Mass (kDa)	Expt. 1^a^^,^^b^	Expt. 2^a^^,^^b^	Information^c^
**Cell Envelopes**						
Q5F6H3	Septum formation inhibitor MafB3	NGO_1585	63.2	0.50	0.51	MafB3, a toxin present on the maf genomic island 1. Contact dependent growth inhibition system. Contains intein domain. endoU ribonuclease. System also encodes immunity protein. Increases fitness in competition assay [53].
A0A0H4IWJ8	Uncharacterized protein	NGO_11150	10.2	4.04	3.61	Pilin. Likely *pilS*. Involved in antigenic variation of PilE through recombination of *pilE* with “silent” copy of *pilS*. *pilS* loci produce sense and antisense RNA. [54, 55]
A0A0H4ISA9	Large *pilS* cassette protein	NGO_10975	24.2	5.04	3.61	Pilin. Involved in antigenic variation of PilE through recombination of *pilE* with “silent” copy of *pilS*. *pilS* loci produce sense and antisense RNA [54, 55].
Q5F851	tRNA-specific adenosine deaminase TadA	NGO_0941	25.9	5.80	4.56	Catalyzes the deamination of adenosine to inosine at the wobble position 34 of tRNA(Arg2). Binds 1 zinc ion per subunit (Uniprot).
A0A0H4IS55	Opacity protein Opa54	NGO_04980	26.2	$\begin{array}{l} 6.47 \end{array}$	6.66	Opacity associated protein (also known as OpaF). Binds to CD66a and CD66e receptors on apical side of T84 cells. Mediates attachment to PMNs and monocytes. Recognized by CEACAM1 and CEACAM5. Receptor specificity studied in strain MS11 [56–59].
A0A0H4IWJ0	Uncharacterized protein	NGO_10985	15.0	7.20	$\begin{array}{l} 7.19 \end{array}$	PilA (FtsY). Signal recognition particle for protein translocation. [60]
**Membrane vesicles**						
Q5F832	Thermonuclease	NGO_0969	24.7	0.64	0.65	Staphylococcal nuclease-like. Allows GC to degrade and escape from NETs. Involved in biofilm structuring [61, 62].
Q5F9Q2	Cysteine synthase	NGO_0340	32.7	0.64	0.67	Member of pathway that synthesizes cysteine. May induce protective immune response in *N*. *meningitidis*, with subpopulation of cells exhibiting surface exposure, despite typically cytoplasmic function [63].
Q5F574	ABC transporter substrate-binding protein	NGO_2056	36.3	1.55	1.95	Thiamine transport system substrate binding protein. Upregulated in response to hydrogen peroxide according to transcriptomics. May be transcriptionally linked to NGO2057 [64].
Q5F5E8	Adhesin complex protein	NGO_1981	20.4	1.57	1.84	Upregulated under anaerobiosis and on exposure to hydrogen peroxide. Adhesin complex protein. Lysozyme inhibitor [64–67].
Q5F9N5	Gly1ORF1	NGO_0358	15.7	1.71	$\begin{array}{l} 1.75 \end{array}$	Knockout of Gly1 was more toxic to fallopian cells [68]. Annotated as chitinase. May enhance virulence by binding to glycoproteins or glycolipids that contain GlcNAc [69].
Q5F6A4	Peptidylprolyl isomerase	NGO_1656	31.5	$\begin{array}{l} 1.73 \end{array}$	1.53	Meningococcal homolog described as cell binding protein, sera from colonized individuals cross-reacted with NMB0345. [70]
Q5F9M5	Uncharacterized protein	NGO_0368	13.8	1.79	1.69	Contains DUF302 domain of unknown function (KEGG)
Q5F7F5	Membrane protein	NGO_1223	24.1	1.81	$\begin{array}{l} 1.64 \end{array}$	Contains DUF3108 domain of unknown function (KEGG)
Q5F5H6	Esterase	NGO_1949	31.4	$\begin{array}{l} 1.82 \end{array}$	1.58	Uncharacterized
Q5F520	MlaC	NGO_2119	21.1	1.83	1.92	Involved in maintenance of lipid asymmetry; periplasmic component of Mla system. Transposon insertion into NGO2119 decreases pilin antigenic variation, DNA repair, and DNA transformation [6, 71]
Q5F8J0	Uncharacterized protein	NGO_0783	18.5	1.90	1.91	Uncharacterized
A0A0H4IVN2	Uncharacterized protein	NGO_11165	13.9	2.13	2.45	Likely PilA (FtsY). Signal recognition particle for protein translocation [60].
Q5F823	Thiol:disulfide interchange protein DsbD	NGO_0978	64.9	2.14	1.88	Uses cytoplasmic thioredoxin to reduce DsbC. Expression is controlled by MisR/MisS two-component system [72].
Q5F5M0	DNA-binding competence protein 2	NGO_1304	10.1	$\begin{array}{l} 2.32 \end{array}$	2.18	Competence protein ComE. DNA binding protein necessary for DNA uptake. Binds without sequence specificity. Deletion does not affect piliation [73].
Q5F9W6	Uncharacterized protein	NGO_0270	17.4	2.42	1.85	Uncharacterized protein. No meningococcal homolog. Closest match *N*. *lactamica* hypothetical protein.
Q5F9Z1	Uncharacterized protein	NGO_0243	19.7	2.54	1.73	Contains DUF2059 domain of unknown function. May have a Correia repeat enclosed element disrupting the coding sequence [74].
Q5FAD1	Pilin assembly protein	NGO_0095	20.1	2.60	2.09	Pilus assembly protein PilP. Ubiquitously expressed in cell envelope, upregulated in response to normal human serum, iron deprivation, anaerobiosis [25].
Q5F8F9	Uncharacterized protein	NGO_0816	10.8	3.32	3.11	Contains DUF4124 domain of unknown function. Hypothetical protein. Closest match is hypothetical protein from *N*. *lactamica*. *N*. *meningitidis* homolog has domain with homology to C-terminal proteolytic portion of LonC protease (KEGG).
A0A0H4ITA9	Uncharacterized protein	NGO_11125	10.0	3.47	6.77	Pilin. Likely PilE. Forms pilus fiber [75].
A0A0H4IS55	Opacity protein Opa54	NGO_04980	26.2	4.38	5.17	Opacity associated protein (also known as OpaF). Binds to CD66a and CD66e receptors on apical side of T84 cells. Mediates attachment to PMNs and monocytes. Recognized by CEACAM1 and CEACAM5. Receptor specificity studied in strain MS11 [56–59].
A0A0H4IVG5	Uncharacterized protein	NGO_07995	13.6	4.96	8.19	Pilin. Likely PilS. Involved in antigenic variation of PilE through recombination of *pilE* with “silent” copy of *pilS*. *pilS* loci produce sense and antisense RNA [54, 55].
A0A0H4IWJ8	Uncharacterized protein	NGO_11150	10.2	5.27	5.47	Pilin. Likely PilS. Involved in antigenic variation of PilE through recombination of *pilE* with “silent” copy of *pilS*. *pilS* loci produce sense and antisense RNA [54, 55]
A0A0H4IWJ0	Uncharacterized protein	NGO_10985	15.0	7.27	6.95	Likely PilA (FtsY). Signal recognition particle for protein translocation [60].

^a^Ratios represent fold change in each experiment.

^b^Ratios for upregulated proteins are shown in red.

^c^Information gathered from bioinformatics tools and literature searches.

Of the 884 CE proteins identified in both replicates (S2 File), 21 and 22 were differentially abundant in the mutant in the first and second experiments, respectively, with 6 common proteins between the two experiments (Fig 9B, left, and Fig 9C). In this group, five proteins showed increased levels and one protein was decreased in the mutant. As expected from our previous proteomic mining of MVs [22], fewer proteins were present in this sub-proteome fraction (568; S3 File). However, the MV protein profile was more dramatically affected by the loss of MlaA than the CE proteome, similar to the qualitative results of coomassie staining (Fig 9A). In the first and second biological replicate, 89 and 81 proteins were differentially expressed in the *mlaA* null strain, respectively, with 23 proteins in common (Fig 9B, right, and Fig 9D). Of the common differentially expressed proteins, 21 increased in abundance in the absence of MlaA, and two proteins showed decreased levels. Additionally, the amounts of NGO2119 (MlaC) were augmented in the MVs isolated from Δ*mlaA*, whereas a corresponding increase was not observed in the CE fraction. Importantly, this result demonstrated that no immediate upstream or downstream effects were introduced to the NGO2116-NGO2124 operon (Fig 2C) during construction of the Δ*mlaA* mutant.

Subsequently, we performed an immunoblot analysis of CE and MV fractions isolated from WT and Δ*mlaA* using available antisera against eight outer membrane proteins including MlaA; BamA, D, and E; LptD; MtrE; TamA; and NGO2054 [25, 76]. As expected, MlaA was not detected in the CE or MVs of Δ*mlaA*. None of the other proteins examined were differentially regulated in the Δ*mlaA* CE or MVs (Fig 9E), which further corroborated the quantitative proteomic investigation (S2 and S3 Files).

One of the proteins that was most highly abundant in the Δ*mlaA* CE compared to WT was the tRNA adenosine deaminase TadA. This essential protein catalyzes an adenosine-to-inosine transition in RNA. A recent report provided the first evidence of mRNA modification in prokaryotes and demonstrated that the modifications were mediated by TadA, although TadA was originally thought to act exclusively on tRNA. *E*. *coli* TadA modifies over 250 proteins, including cell envelope proteins BamA, TamA, and PldA [77]. The tyrosine-to-cysteine transitions induced by TadA modification altered the toxicity of one of the target proteins, the toxin HokB [77], which may suggest that the biological activities of gonococcal TadA substrates are affected by TadA upregulation in the Δ*mlaA* mutant.

The increased presence of protein products with homology to *pilS* (NGO10975, NGO11150, and NGO07995; Table 3) in the mutant CE and MVs may indicate that pilin antigenic variation is enhanced in *mlaA* null bacteria. *pilS* cassettes are transcriptionally silent loci involved in antigenic variation of PilE through RecA-mediated recombination [54]. Also upregulated in the Δ*mlaA* CE and MVs was the opacity-associated protein Opa54 (NGO04980; OpaF). Opa proteins interact with members of the carcinoembryonic antigen-related cell adhesion molecule (CEACAM) family of receptors to mediate bacterial attachment to epithelial cells, neutrophils, and monocytes [56–59].

In addition to pilus-related proteins, several other potential virulence factors within the MVs of the Δ*mlaA* mutant were elevated. Across the two experiments, the *Neisseria* adhesin complex protein (ACP) was present in higher quantities in the mutant MVs by an average of 1.7-fold. ACP is involved in meningococcal adhesion to human cells [67] and possesses a secondary function as a lysozyme inhibitor [66]. Originally discovered in a screen for gonococcal hemolysins [68], Gly1ORF1 (NGO0358) was also increased 1.7-fold in the Δ*mlaA* MVs. The *ngo0358* locus is annotated as a putative chitinase. While overexpression of *gly1ORF1* has not been studied in *N*. *gonorrhoeae*, chitinases play roles in the virulence of other bacterial species by binding to the N-acetyl-glucosamine moiety of glycolipids and glycoproteins [69].

Together, our studies are the first to address the expression and function of MlaA in gonococcal physiology and pathogenesis, revealing that the loss of MlaA not only affects colony morphology, CE and MV subproteomes, resistance to certain antimicrobials, and MV formation, but also increases the fitness of *N*. *gonorrhoeae* under *in vitro* conditions relevant to infection and in the murine female genital tract. Cumulatively, our findings highlighted a new mechanism of *N*. *gonorrhoeae* pathogenesis and a better understanding of the function(s) of MlaA in this clinically relevant pathogen.

## Discussion

The asymmetry of the Gram-negative outer membrane is responsible for its formidable barrier function. When this asymmetry is disrupted, phospholipids accumulate in the outer leaflet and the cell becomes more vulnerable to both hydrophilic and lipophilic antimicrobial compounds [3, 6]. Thus, bacteria have developed several systems to maintain the asymmetry of the outer membrane. Two systems, PagP and PldA, destroy outer-leaflet phospholipids, while the Mla system removes intact phospholipids and re-integrates them into the inner membrane [6]. MlaA, located within the outer membrane in complex with OmpF, forms a channel through which the head groups of phospholipids are able to travel [10]. Our bioinformatics searches did not identify a PagP homolog in the gonococcal genome, indicating that *N*. *gonorrhoeae* relies solely on PldA and the Mla system to maintain phospholipid homeostasis. This aspect of bacterial physiology has not been well studied in *Neisseria*, and reports on PldA mainly focus on its role during autolysis [78–80].

Bioinformatic analyses revealed the existence of two separate classes of MlaA homologs (Fig 2A). *N*. *gonorrhoeae* MlaA is a member of the class composed primarily of proteins lacking a lipoprotein signal peptide, with the exception of *K*. *pneumoniae* and *F*. *tularensis*. *In vivo* assessments with MlaA (VacJ) knockout strains revealed virulence defects in members of the lipoprotein-containing phylogenetic cluster: *S*. *flexneri*, *H*. *parasuis*, and *S*. *enterica* enterica serovar Typhimurium [11, 13–15]. In contrast, *P*. *aeruginosa* deficient in VacJ, which lacks a lipoprotein signal peptide, was more virulent than WT bacteria [12]. The opposing results suggest that MlaA may play a different moonlighting role(s) in certain aspects of pathogenesis for bacteria, depending on its association with the CE.

We also provide the first report of differences between the genetic location and organization of *mlaA* in *N*. *gonorrhoeae*, *N*. *meningitidis*, and *N*. *lactamica* compared to those in other bacteria. In the *Neisseria* species examined, *mlaA* appears to be part of a polycistronic operon composed of the other components of the Mla system, while *mlaA* is physically separated from the other Mla members in *E*. *coli* (Fig 2C and S2 Fig). Therefore, *Neisseria* may employ a different regulation strategy for MlaA. Due to its genomic organization, complementation of individual *mla* components is technically challenging, if not entirely impossible, without a greater understanding of *mla* system regulation. This hindrance was illustrated by low levels of MlaA in the Δ*mlaA*/P_lac_::*mlaA* strain regardless of the high amount of the inducer used (Fig 3E, S4 Fig). The little amount of MlaA was sufficient to return mutant colonies to nearly WT size during polymyxin B exposure (Fig 6B, 6D and 6E), but was not able to complement the competitive infection phenotype (Fig 8A and 8B; S6 Fig). The difficulty of complementing knockouts in a polycistronic operon, even in a fairly well-studied system in which many of the regulatory elements are already known, has been recognized [81–83]. For instance, in the soil bacterium *Agrobacterium tumefaciens*, a virulence plasmid, pTi, carries an operon composed of 11 *virB* genes that are each essential for tumor formation in plants [84–86]. A complementation study to examine each member of the *virB* operon revealed additional regulatory requirements for six of the 11 genes, including switching the constitutive (in the *A*. *tumefaciens* system) *lacZ* promoter for the native *virB* promoter for VirB1 and VirB2, adding 55 or 230 bp of upstream sequence for VirB6 and VirB9, or co-expressing VirB7 and VirB8 on the same complementation plasmid [86].

Mla is a relatively recently discovered protein complex and limited information is available regarding expression of the Mla components, including MlaA [10, 13]. To address this gap, we examined MlaA expression patterns throughout the growth of WT *N*. *gonorrhoeae* in liquid media, during exposure to host-relevant conditions, and in a panel of diverse *Neisseria* (Figs 4–5). We demonstrated that the amount of MlaA was decreased in the absence of iron and increased during anaerobic growth (Fig 5C and 5G). The down-regulation in the absence of iron is consistent with the Fur-dependent *vacJ* transcription pattern in *H*. *influenzae* during *in vitro* growth and *in vivo* in a mouse model of nasopharyngeal colonization [13]. While MlaA has not been described as part of the *N*. *gonorrhoeae* iron regulon, transcription of MlaF (NGO2116) decreases under iron restriction in a Fur-dependent manner [87], which suggests that the entire Mla system may be under Fur transcriptional control in the gonococcus. Our investigation into the effects of Fur on MlaA suggest that Fur’s influence has diminished over the intervening five genes. Free iron concentrations are maintained in the attomolar range (10^−18^ M) in bodily fluids to prevent microbial growth, and infection induces lactoferrin secretion to further restrict iron availability [88], including in the reproductive tract. Transcriptomic studies of gonococci isolated from active infections have shown that *mlaA* is transcribed during cervical infections after recent exposure to an infected male partner, and *mlaA* transcripts are typically lower during infection than during growth in defined medium [89]. Together, these lines of evidence suggest that the downregulation of *mlaA* in response to iron restriction, but not the complete abrogation of expression, is part of the infection strategy employed by the gonococcus.

Overexpression of the phospholipase PldA in the Δ*mlaA* background reduced MV formation but strongly enhanced bacterial vulnerability to polymyxin B (Figs 7C and 6B and 6C, respectively). An identical experiment has not been performed in *E*. *coli*. Differences in phospholipid composition between *N*. *gonorrhoeae* and *E*. *coli* explain our results (Table 4). *E*. *coli* membranes contain ~87% phosphatidylethanolamine (PE) and 4.7% phosphatidylglycerol (PG) in late-stage cultures [90], while PE and PG comprise 69% and 19% of the membrane of *N*. *gonorrhoeae*, respectively [91]. In both species, PldA predominantly destroys PE [79, 92]. In contrast, the Mla system does not appear to exhibit a preference for specific phospholipids [93]. In *N*. *gonorrhoeae*, only the Mla system and PldA contribute to outer membrane asymmetry as no PagP homolog exists. Thus, in our proposed model, in the absence of MlaA, with native PldA levels, the overall phospholipid composition is largely preserved [13], but the outer membrane will contain phospholipids that have invaded the CE outer leaflet (Fig 1B). PldA dimerization and activation will likely increase upon detection of perturbed phospholipid homeostasis [8], although evidence from *E*. *coli* suggests that PldA expression will not be altered in the absence of membrane stress [6]. The enhanced activation of endogenous PldA levels may alter the phospholipid profile slightly. However, upon PldA overproduction in the Δ*mlaA* mutant, the substrate preference of PldA towards PE will result in a higher abundance of PG within the surface of *N*. *gonorrhoeae* (Fig 1C; Table 4). Polymyxin B preferentially targets PG [94, 95]. Gonococci overexpressing PldA in the absence of MlaA are therefore more sensitive to the antimicrobial peptide.

**Table 4:** Phospholipid composition of *N*. *gonorrhoeae* and *E*. *coli* membranes.

Phospholipid	*N*. *gonorrhoeae* [91]	*E*. *coli* [90]
Phosphatidylethanolamine (PE)	69%	87%
Phosphatidylglycerol (PG)	19%	4.7%
Cardiolipin	0.8%	7.1%
Phosphatidylcholine (PC)	11%	N.D.^a^
Phosphatidic acid	N.D.^a^	0.8%

^a^N.D., Not detected

Downregulation and deletion of VacJ in *H*. *influenzae* and *V*. *cholerae* increased MV formation, and accumulation of phospholipids in the outer leaflet has been proposed as a general mechanism of MV biogenesis [13]. Corroborating this suggestion, the *N*. *gonorrhoeae* Δ*mlaA* strain produced significantly more MVs than WT bacteria and PldA overproduction partly reduced their amounts (Fig 7C). However, dramatic increases in MV release for both WT and Δ*mlaA* bacteria under polymyxin B stress (Fig 7C) provided evidence that invading phospholipids are not the primary factor in MV biogenesis.

Why do we observe increased fitness of the Δ*mlaA* during competitive infection in the surrogate host? We propose that it is due to the altered CE and MV protein contents (Fig 9A). Quantitative proteomics of these fractions revealed alterations in proteins with significant implications in bacterial physiology and virulence (Fig 9B, 9C and 9D). Among the most upregulated proteins in the Δ*mlaA* CE was an essential enzyme, TadA, which edits mRNA without changing its expression and recodes protein sequences, potentially affecting their function and cell physiology [77]. Our proteomic profiling revealed a previously unrecognized link between phospholipid homeostasis and RNA editing, which should be examined in future studies, in addition to the role of TadA-mediated protein modification in bacterial pathogenesis. Further, several adhesins were also elevated in the CE and MV fractions of the Δ*mlaA* mutant (Fig 9C and 9D, Table 3). Of these, adherence mediated by pili and Opa proteins is host-restricted, although a CEACAM receptor-independent advantage is observed for Opa-positive gonococci in the mouse model [51, 96]. Elevated Opa levels in the Δ*mlaA* mutant may therefore have contributed to the mutant’s enhanced fitness. Heightened pilin antigenic variation in the absence of MlaA may also have played a role. However, one or more of the other proteins may contribute more substantially to the increased fitness of the mutant in the mouse model (Fig 8B), possibly through elevated ACP acting as a lysozyme inhibitor [97, 98] within the MVs [56–59, 67]. MVs have been shown to act as decoys against components of the innate immune system, by adsorbing antimicrobial peptides, for example [99], and are often packages for virulence factors [100]. The Δ*mlaA* mutant appears to exploit both of these functions, as illustrated by the presence of heightened levels of ACP within MVs, which could protect the bacteria from lysozyme attack [66, 97]. We have not assessed lysozyme sensitivity in the Δ*mlaA* mutant, as two gonococcal lysozyme inhibitors, ACP and SliC, are responsible for lysozyme resistance and compensate for each other *in vitro* [97, 98]. However, lack of SliC was sufficient to significantly impact bacterial fitness in the mouse genital tract [97]. Thus, the loss of MlaA results in multifactorial alterations to gonococcal CE and MV protein composition that likely act in concert to enhance the fitness of the Δ*mlaA* mutant in the mouse model. The primary function of MlaA is in cell envelope biogenesis and homeostasis. However, alterations to the MlaA cellular pool in response to host conditions would modulate the CE and MV protein composition, as well as the amount of blebs released.

Our study presents MlaA as an intriguing protein with an inverse effect on gonococcal pathogenesis. We propose that *N*. *gonorrhoeae* adjusts virulence through changes in MlaA levels in response to iron availability in the host, as both the human [88] and murine [101] genital tracts are iron-restricted. Downstream effects of MlaA depletion involve alterations to the CE and MV protein composition, including elevated levels of known and potential virulence factors, as well as the possibility of post-translational protein modification. It is exciting to consider that other host/bacterial effectors may also be involved in MlaA-triggered MV biogenesis and in fine-tuning the bacteria-host interaction.

## Materials and methods

### Bacterial strains and growth conditions

For this study, laboratory strain FA1090 [102] and contemporary clinical isolate WHO X [26] were used as wild type (WT) strains. Isogenic null mutants of *ngo2121* were previously constructed in both strain backgrounds by replacement of the locus with the kanamycin resistance cassette [22, 23], and a complemented strain with *ngo2121* under the control of the P_*lac*_ promoter was also previously constructed in the FA1090 strain background [23]. For this study, we also generated a conditional knockout of the Fur transcriptional regulator, Δ*fur*/P_lac_::*fur*, described below. To analyze expression of MlaA in clinical strains, we used a panel of isolates collected from two public health clinics in Baltimore from 1991 to 1994 (LGB1, LG14, LG20, LG26); the Public Health-Seattle & King County Sexually Transmitted Disease clinic from 2011 to 2013 (UW01-UW13); and the 2016 WHO reference strains [25]. Frozen stocks of *N*. *gonorrhoeae* strains stored at -80 °C were streaked onto gonococcal base agar solid medium (GCB, Difco) supplemented with Kellogg’s supplements I and II diluted 1:100 and 1:1,000, respectively [25]. After incubation for 18–20 h at 37 °C in a 5% CO_2_ atmosphere, colonies were subcultured onto GCB. Transparent piliated cells were used for transformation, while transparent, non-piliated variants were used for all other experiments. Unless otherwise specified, after 18–20 h of subsequent culturing as described above, colonies were collected from solid medium with a polyester-tipped sterile applicator (Puritan) and suspended to a final OD_600_ of 0.1 in gonococcal base liquid medium (GCBL) supplemented with 0.042% sodium bicarbonate and Kellogg’s supplements as above. Bacteria were then cultured at 37 °C with agitation (220 rpm) for 3 h, diluted to a final OD_600_ of 0.1 in supplemented GCBL, and cultured as previously.

*E*. *coli* NEB5α was used for genetic manipulations, while *E*. *coli* BL21(DE3) was used for heterologous expression of MlaA and DsbA for protein purification. *E*. *coli* was maintained at 37 °C in Luria-Bertani (LB, Difco) media or LB agar supplemented with the appropriate antibiotic. Concentrations of antibiotics used are as follows: *N*. *gonorrhoeae* kanamycin (40 μg/mL for FA1090, 50 μg/mL for WHO X) and erythromycin (0.5 μg/mL for FA1090, 4 μg/mL for WHO X); *E*. *coli* kanamycin (50 μg/mL).

### DNA manipulations

MlaA_120-277_ was amplified from purified FA1090 genomic DNA using primers MlaA-trunc-F, TTCATCCATATGCCCGACAATAAAAACACTTTGG and MlaA-trunc-R, TTCATCCTCGAGGGGTTGTGTTCCAGGTTG. The PCR product was digested with NdeI and XhoI restriction enzymes (New England Biolabs; restriction sites are underlined), inserted into similarly-digested pET28a to place a 6 × His-tag on the C-terminus, and transformed into *E*. *coli* BL21(DE3).

The gene encoding *dsbA* missing the signal peptide was amplified using primers DsbA-f, GACTCCATGGTGACGGAAGGGGAAGACT and DsbA-r, GACTAAGCTTCGGCTATTTCTGTACAGCAG. The subsequent PCR product was digested with NcoI and HindIII, as indicated, and ligated into similarly cut pRSF-NT to create a tobacco etch virus protease-cleavable C-terminal-6 × His-tagged fusion. PldA was amplified using primers PldA-F, GATCTTAATTAAAAAATGCCGTTTGAAAACCAATATG and PldaA-R, CGGTATTCAGATGCCGTC. PCR product was digested with PacI, ligated into pGCC4 digested with PacI and PmeI (New England Biolabs; PacI restriction site underlined), and transformed into *E*. *coli* NEB5α. After sequence verification, pGCC4-PldA was transformed into piliated Δ*mlaA* as previously [22].

The conditional *fur* knockout was created using a strategy described previously [25]. An additional copy of the *fur* gene was initially placed under the control of an IPTG-inducible promoter within the intergenic region between *lctP* and *aspC* in the FA1090 chromosome. *fur* was subsequently replaced in its native chromosomal locus with the nonpolar kanamycin resistance cassette. Specifically, the *fur* gene was amplified using primers fur-F, GATCTTAATTAATTACAGGGATATTGAATATTATGGAAAAATTC and fur-R, ACAACAAACCGTCCGGAT. The PCR product was cut with PacI and ligated into PacI/PmeI cut pGCC4 to create pGCC4-fur. The resulting plasmid was used for transformation of FA1090 to create FA1090P_lac_::*fur*. To generate the knockout plasmid, pNEB193 was linearized by PCR using primers pNEB-F, GTTTAAACCTGCAGGCATGCAAG and pNEB-R, TCTAGACTTAATTAAGGATCCGGCG. One thousand bp upstream and downstream of *fur* were amplified using primers fur-up-F, CGCCGGATCCTTAATTAAGTCTAGATTTCGGCGGCGGCGC; fur-up-R, TCTGAGCGGGACTCCCTGTAAAATAATAGACGCTATAATACGCAATTTCAG; fur-down-F, GTGAAGCTAGCTATCCGGACGGTTTGTTGTTCAG; and fur-down-R, AGCTTGCATGCCTGCAGGTTTAAACTTTCCAATTTTATAGTGGATTAAATTTAAACCGGTACGG. The kanamycin resistance cassette was amplified using primers kan-F, TATTTTACAGGGAGTCCCGCTCAGAAGAACTCG and kan-R, AAACCGTCCGGATAGCTAGCTTCACGCTGCC. All fragments were purified and assembled using NEBuilder HiFi DNA Assembly Master Mix (NEB). pNEB193-Δfur was linearized with ScaI and used to transform FA1090P_lac_::*fur*. Clones were selected on solid medium supplemented with kanamycin and 0.1 mM IPTG and verified by PCR.

All plasmid insert sequences were confirmed by the Oregon State University Center for Genome Research and Biocomputing.

### Protein purification

Truncated, recombinant 6 × His tagged MlaA (MlaA_120-277_) was expressed in *E*. *coli* BL21(DE3) harboring pET28-MlaA_120-277_. An overnight culture was used to inoculate 1.5 L LB medium supplemented with kanamycin. Protein overproduction was induced with 500 mM IPTG when cultures had reached OD_600_ ~0.5. After 3 h of growth at 37 °C, cells were pelleted by centrifugation (6,000 rpm for 20 min at 4 °C) and stored at -80 °C until use. Pellets were resuspended in lysis buffer (20 mM Tris pH 8.0, 500 mM NaCl, 10 mM imidazole, 1% Triton-X) in which a Pierce protease inhibitor mini tablet (ThermoFisher Scientific) had been dissolved. Cells were lysed by six passages through a French Press pressure cell at 1,200 psi. Crude lysate was clarified by centrifugation, incubated with DNAseI, and filtered through a 0.45 μm filter. Protein was purified on a Bio-Rad NGC scout system through a Bio-Rad Bio-Scale Mini Nuvia IMAC cartridge (Bio-Rad). Non-specifically bound proteins were washed off the column by 10 column volumes of wash buffer (20 mM Tris pH 8.0, 500 mM NaCl, 40 mM imidazole, 1% Triton-X). Protein was eluted over a 40–250 mM imidazole gradient and fractions were monitored for protein purity by SDS-PAGE. Triton was removed from protein solution by 2 h of end-over-end incubation with Bio-Rad Bio-Beads SM-2 adsorbent polystyrene beads (Bio-Rad) at room temperature. Glycerol was added to a final concentration of 10% and protein was stored at -80 °C.

For the overproduction of DsbA, 1 L of LB supplemented with kanamycin was inoculated with *E*. *coli* BL21(DE3) carrying pRSF-*dsbA*. Protein overproduction was induced by the addition of 1 mM IPTG when the culture reached an OD_600_ of about 0.5. After 3 h of growth at 37 °C, bacterial cells were pelleted by centrifugation at 6,000 × g for 20 min at 4 °C and cell pellets were stored at -80 °C until used. Recombinant DsbA was purified under native conditions using the NGC Scout Chromatography system (Bio-Rad). Bacterial cells were resuspended in lysis buffer (20 mM Tris pH 8.0, 500 mM NaCl, 10 mM imidazole) and lysed by five passages through a French pressure cell at 1,200 psi. Cell lysates were clarified by centrifugation and loaded onto a Bio-Scale Mini Profinity IMAC cartridge (Bio-Rad). Loosely bound proteins were removed with 10 column volumes of wash buffer (20 mM Tris pH 8.0, 500 mM NaCl, 40 mM imidazole) and DsbA was eluted with a 40–250 mM imidazole gradient. To remove the 6 × His tag the eluted DsbA was incubated overnight at 4° C with TEV protease at a 1:20 ratio. The proteins were concentrated to 5 mL using a Vivaspin 20 centrifuge concentrator (GE HealthCare). Proteins were subjected to size exclusion chromatography using HiLoad 16/600 Superdex 75 pg (GE HealthCare) and phosphate buffered saline (PBS) as running buffer. Fractions containing DsbA were concentrated using a Vivaspin 20 centrifuge concentrator (GE HealthCare).

### Antisera preparation

Polyclonal rabbit antisera were prepared by Pacific Immunology using about 2 mg of purified MlaA_120-277_ or DsbA. A 13-week antibody production protocol was approved by IACUC Animal Protocol #1 and the National Institute of Health Animal Welfare Assurance Program (#A4182-01), and was performed in a certified animal facility (USDA 93-R-283).

### SDS-PAGE and immunoblotting

Samples as indicated in the text were standardized by OD_600_ values (whole cell lysates) or by protein concentration (subcellular fractionation samples, i.e. C/P, CE, MV, and SS fractions). Standardized samples were separated by 1 dimensional sodium dodecyl sulfate–polyacrylamide gel electrophoresis (SDS-PAGE) on 4–12% Novex NuPAGE (ThermoFisher Scientific) or 4–15% Bio-Rad Criterion TGX (Bio-Rad) protein gels. Proteins were visualized by colloidal coomassie G-250 staining. For immunoblotting, proteins were transferred to Trans-Blot Turbo nitrocellulose 0.2 μm membranes (Bio-Rad) using a Trans-Blot Turbo transfer system (Bio-Rad) at 25 V for 7 min. Membranes were blocked in blocking buffer (5% skim milk in phosphate buffered saline with 0.1% Tween-20 [PBST]) for 1 h at room temperature on a rocking table or at 4 °C overnight. Primary antisera were diluted in blocking buffer as follows: α-MlaA: 1: 5,000; α-TbpB: 1: 1,000 [25]; α-MtrE: 1: 5,000 [25]; α-BamA: 1: 10,000 [25]; α-BamD: 1: 20,000 [25]; α-LptD: 1: 5,000 [25]; α-TamA: 1: 10,000 [25]; α-NGO2054: 1: 10,000 [25]; α-SurA: 1:10,000 [46]; α-DsbA: 1:10,000; and α-Zwf: 1: 10,000 [49]. Membranes were incubated in horseradish peroxidase-conjugated goat anti-mouse (MtrE) or–rabbit (all other antigens) secondary antibody diluted 1:10,000. Blots were developed in Clarity Western ECL Substrate (Bio-Rad) and imaged on a ChemiDoc MP (Bio-Rad). Densitometry analyses were performed with Image Lab software (Bio-Rad).

### Exposure to host-relevant conditions

FA1090 (WT) and Δ*mlaA* bacteria cultured as above were collected from rapidly-growing liquid cultures, diluted to OD_600_ = 0.2, serially diluted, and spotted on GCB plates supplemented with 7.5% NHS or 5 μM of the iron chelator deferoxamine mesylate (desferal). Inoculated plates were incubated at 37 °C in a 5% CO_2_ environment for 22 h or anaerobically at 37 °C in the presence of 1.2 mM sodium nitrite as a terminal electron acceptor, and either 0, 5, 10, 20, or 25 μM desferal for immunoblotting, for 48 h. The anaerobic environment was generated using BD GasPaks (BD) in an anaerobic jar. Colonies were counted for colony forming unit (CFU)/mL enumeration. Bacteria were collected from plates for immunoblotting analysis.

Desferal titration was performed in liquid media. Non-piliated FA1090 WT or Δ*mlaA* were suspended to OD_600_ = 0.1 in GCBL supplemented with Kellogg’s supplement I and sodium bicarbonate, but without Kellogg’s supplement II. Strains were cultured at 37 °C for 3 h with agitation (220 rpm). Cultures were diluted to OD_600_ = 0.1 in media supplemented with Kellogg’s supplement I and sodium bicarbonate and split. Daughter cultures were supplemented either with Kellogg’s supplement II or Desferal (5 to 25 μM). Suspensions were cultured at 37 °C with agitation for 6 h. Every hour, including 0 h, samples of each culture were serially diluted. Five microliters of each dilution were spotted onto a GCB plate, which was subsequently cultured at 37 °C for 18–20 h at 5% CO_2_. Colonies were counted for CFU/mL enumeration after incubation. Samples of WT and Δ*mlaA* liquid cultures with different desferal concentrations were taken at the 6 h timepoint and subjected to immunoblot analysis.

### MlaA Fur regulation assessment

MlaA regulation by Fur was evaluated using the conditional Fur knockout strain Δ*fur*/P_lac_::*fur*. Initial liquid cultures of WT FA1090, Δ*mlaA*, and Δ*fur*/P_lac_::*fur* were prepared without Kellogg’s supplement II. Fur expression was induced by the addition of 0.1 mM IPTG. After three hours of growth, Δ*fur*/P_lac_::*fur* cultures were washed by centrifugation at 5,000 × *g* for 5 minutes to remove IPTG, the supernatant was decanted, and bacteria were resuspended in fresh medium without IPTG. All strains were back diluted to an OD_600_ of 0.1 and supplemented with either Kellogg’s supplement II or 25 μM desferal. Fur expression was induced at different levels by the addition of 10, 50, or 100 μM IPTG under both iron-deplete and–replete conditions. Bacteria were cultured for 6 h at 37 °C with shaking (220 rpm), and growth was monitored every hour by OD_600_ measurement. At the experimental endpoint, samples of each culture were collected and centrifuged at 6,000 × *g* for 10 minutes. The supernatant was discarded and pellets were prepared for SDS-PAGE and immunoblotting analysis.

### Serum sensitivity

Assessment of bacterial serum sensitivity was essentially as previously described [37]. Non-piliated strains as indicated in the text were suspended to OD_600_ = 0.1 in supplemented GCBL and cultured for 3 h at 37 °C with shaking (220 rpm). Bacterial cultures were suspended to OD_600_ = 0.05 in sterile PBS, then diluted 1:1000 in Eagle’s minimal essential medium (EMEM). Seventy microliters of this suspension, corresponding to approximately 10^3^ CFU, was added to 70 μL NHS (Quidel, San Diego, CA; Ref# A113), heat-inactivated NHS (inactivated at 56 °C for 30 min), or EMEM alone, for final serum concentrations of 0% or 50% in a 96 well microtiter plate (initial testing indicated that bacterial survival was not altered in the presence of 8%, 10%, 12%, or 24% NHS). Plates were incubated at 37 °C in 5% CO_2_ for 1 h, after which 20 μL from each well were spotted onto a GCB plate. CFU/mL were enumerated following 18–20 h incubation as above.

### Exposure to human defensin

HBD1 (AnaSpec Inc. 510791–9560) was solubilized to 100 μM in 0.01% acetic acid. Rapidly growing liquid cultures of WT and Δ*mlaA* bacteria were diluted to 10^5^ cells/mL, and 45 μL of this suspension were transferred to a 1.5 mL tube. Volumes were adjusted to 50 μL with either GCBL, 0.01% acetic acid (vehicle), or HBD1 (final concentration, 10 μM). Bacteria were incubated for 3 h at 37 °C, serially diluted, and spotted onto GCB. CFUs were enumerated following 22 h incubation, and the relative survival was calculated. Experiments were performed on at least four independent occasions.

### Etest antimicrobial sensitivity testing

Antimicrobial susceptibility was assessed by Etests according to manufacturer’s instructions. Briefly, non-piliated colonies of FA1090 or WHO X WT, Δ*mlaA* bacteria in either strain background, or Δ*mlaA*/P_lac_::*pldA* in the FA1090 knockout background were collected from GCB plates and suspended in brain heart infusion liquid medium to a turbidity equivalent to that of a 0.5 McFarland standard. Suspensions were spread on 150 mm tissue culture dishes containing 50 mL GCB solid medium (~4 mm thick), and test strips were laid on the surface of the plates. 0.5 mM IPTG was added to Δ*mlaA*/P_lac_::*pldA* plates. MICs were measured after approximately 22 h of incubation as above. Experiments were performed on three occasions and consensus MICs from at least two out of the three replicates are presented.

### Sensitivity to polymyxin B

FA1090 WT, Δ*mlaA*, Δ*mlaA*/P_lac_::*mlaA*, and Δ*mlaA*/P_lac_::*pldA* were suspended to OD_600_ = 0.1 in GCBL supplemented as above and cultured for 3 h at 37 °C with agitation (220 rpm). Cultures were subsequently diluted to an OD_600_ of 0.1 in supplemented GCBL and cultured at 37 °C with agitation for 2 h. Bacteria were serially diluted and spotted on plates with or without 800 U/mL polymyxin and 0, 0.05, 0.1, or 0.5 mM IPTG. Plates were cultured at 37 °C in a 5% CO_2_ environment for approximately 18 h. Overall colony morphology was documented with a Chemi-Doc MP (Bio-Rad). Colonies were counted to determine overall survival under polymyxin B exposure. Images of colonies were taken with a Zeiss AxioObserver.D1 microscope at 10× magnification 0.25 Phase Contrast 1 and at 2.5× magnification 0.06 Phase Contrast 1. Colony diameters from images taken at 2.5× magnification were measured using ImageJ software. Results were similar for all induction levels. For clarity, % survival data is presented only for the 0.5 mM IPTG level. Microscopy for the complemented strain was performed with 0.1 mM IPTG.

### LOS isolation and silver staining

LOS was isolated from WT, Δ*mlaA*, Δ*mlaA*/P_lac_::*mlaA*, and Δ*mlaA*/P_lac_::*pldA* using a method described previously [49]. Strains were harvested from either standard liquid cultures or cultures containing 100 U/mL polymyxin B by suspension in 1.5 mL GCBL to an OD_600_ of 0.2 and centrifugation at 15,000 × *g* for 1.5 min. The expression of MlaA in the Δ*mlaA*/P_lac_::*mlaA* strain was induced with 0.5 mM IPTG, and Δ*mlaA*/P_lac_::*pldA* was cultured either with or without 0.5 mM IPTG, as indicated. Cell pellets were resuspended in 50 μL lysis buffer (2% SDS, 4% β-mercaptoethanol, 10% glycerol, 1 M Tris pH 6.8, and 0.01% bromophenol blue) and boiled at 100 °C for 10 minutes to lyse bacteria. After samples were allowed to cool to room temperature, proteins were digested by proteinase K (25 μg in 10 μL lysis buffer) at 60 °C for 1 h. SDS-PAGE was performed on isolated LOS using an 18% gel, and LOS was visualized by silver staining [49].

### Supernatant protein profiling

Membrane leakiness was assessed as previously described [23], using filtered supernatants from mid-logarithmic liquid cultures. Soluble supernatant proteins were precipitated with a pyrogallol red-molybdate-methanol procedure after DNAse I treatment. Membrane vesicles were not separated prior to precipitation. Precipitated proteins, standardized by OD_600_ values of the source cultures, were separated by SDS-PAGE and analyzed by immunoblotting. Whole cell lysates of source cultures were analyzed simultaneously.

### Electron microscopy

Strains indicated in the text were cultured in GCBL as above, either with or without 100 U/mL polymyxin B, until approximately mid-logarithmic growth (OD_600_ of 0.5). The Δ*mlaA*/P_lac_::*mlaA* and Δ*mlaA*/P_lac_::*pldA* strains were cultured in the presence (both strains) or absence (Δ*mlaA*/P_lac_::*pldA* only, as indicated) of 0.5 mM IPTG. Bacteria were collected by centrifugation at 4000 × *g* for 3 min, washed twice and resuspended with PBS sterilized by filtration through a 0.1 μm filter. 2.5 μL of suspension were spotted onto 300 mesh copper grids and cells were allowed to attach to the grid for 15 min before excess PBS was removed. Cells were negatively stained with phosphotungstic acid and visualized with a FEI Helios NanoLab 650 electron microscope housed at the Oregon State University Electron Microscopy Facility. Experiments were performed at least twice, and representative micrographs are presented.

### Subcellular fractionation

C/P, CE, MV, and SS fractions were isolated as described previously [22, 25]. Briefly, strains as indicated in the text were suspended in 500 mL GCBL supplemented as above and cultured at 37 °C with agitation (220 rpm) until cultures reached OD_600_ of ~0.8. Where indicated in the text, cultures were also supplemented with 100 U/mL polymyxin B. PldA overproduction was induced by the addition of 0.5 mM IPTG. Supernatants were separated from bacteria by low speed centrifugation and filtration and treated with DNaseI and protease inhibitors. Naturally released MVs were isolated by high-speed ultracentrifugation. Supernatants of MV isolation contain soluble supernatant proteins, which were precipitated by incubation with 15% trichloroacetic acid at 4 °C for 1 h and centrifugation at 14,000 × *g* for 20 min at 4°C. Precipitated proteins were washed with ice-cold acetone and centrifuged at 14,000 × *g* for 20 min at 4 °C. MV and SS fractions were resuspended in PBS + 0.8% SDS for SDS-PAGE, immunoblotting, and quantitative proteomics analyses as indicated. The CE was isolated by cold sodium carbonate extraction and ultracentrifugation [22, 25], and suspended in PBS + 0.8% SDS. Samples of supernatants from CE isolation were collected for the cytoplasmic/periplasmic fraction.

### Quantitative proteomics

The quantitative proteomics studies were performed as we described previously [103]. Briefly, CE or MV were isolated from WT and Δ*mlaA* bacteria when the OD_600_ of each culture reached 0.6–0.8, as described above. The total protein amount in each CE and MV fraction was assessed using a Protein Assay Kit (Bio Rad) and were subjected to labeling with TMT6plex reagent (Thermo Scientific) according to manufacturer recommendations. Eighty μg of proteins were placed in a 1.5 mL tube and the volume was adjusted to 100 μL using 100 mM triethylammonium bicarbonate (TEAB) buffer. Proteins were reduced by addition of 11.3 mM tris(2-carboxyethyl)phosphine hydrochloride (TCEP) and incubation at 55 °C for 1 h, and alkylated by addition of 20.1 mM iodoacetamide for 30 min in the dark. Proteins were precipitated overnight in 90% acetone at -20°C, spun down at 15,000 × *g* for 10 min at 4°C and washed once with ice cold acetone. Protein pellets were resuspended in 100 μL of 100 mM TEAB and digested with trypsin at a 1:40 ratio overnight at 37°C. TMT Reagents were reconstituted in 41 μL of acetonitrile (ACN) and were added to the digested samples. Wild type CE samples were labeled with reagent TMT126 and TMT128 for replicates 1 and 2, respectively. CE proteins isolated from Δ*mlaA* were labeled with TMT127 and TMT129 for replicates 1 and 2, respectively. To label the MV proteins, TMT128 and TMT130 were used for wild type and TMT129 and 131 for Δ*mlaA*. The reaction was allowed to proceed for 1 h at room temperature and was quenched by addition of 8 μL of 5% hydroxylamine. Samples were pooled and subsequently fractionated by strong cation exchange (SCX) with a Paradigm (Michrom Biosciences) HPLC using 5 mM potassium phosphate monobasic in 30% ACN/70% water (v/v) pH 2.7 (buffer A) and 5 mM potassium phosphate monobasic in 30% ACN/70% water (v/v) pH 2.7 with 500 mM potassium chloride (buffer B) as the mobile phases. The sample was brought up in 200 μL buffer A. Peptides were separated over 60 min with a 2.1 mm x 100 mm Polysulfoethyl A column (PolyLC) at a 200 μL/min flow rate using the following separation profile: hold 2% B for 5 min, 2% to 8% B in 0.1 min, 8% to 18% B in 14.9 min, 18% to 34% B in 12 min, 34% to 60% B in 18 min, 60% to 98% B in 0.1 min and hold for 10 min. We collected 1 min fractions in 96-well microtiter plates. Twelve pools were generated from 60 fractions, and the pools were dried with a speed vac. Oasis HLB 1cc cartridges were subsequently used to desalt the samples. Cartridges were initially washed with 70% ACN/0.1% trifluoroacetic acid (TFA), then equilibrated with 0.1% TFA. Fractionated samples were hydrated in 0.1% TFA to load onto the cartridge, washed with 0.1% TFA, eluted in 1 mL 70% ACN/0.1% TFA, and dried by vacuum centrifugation.

Desalted samples were subsequently analyzed by LC/ESI MS/MS with a Thermo Scientific Easy-nLC II (Thermo Scientific, Waltham, MA) nano HPLC system coupled to a hybrid Orbitrap Elite ETD (Thermo Scientific, Waltham, MA) mass spectrometer at the Proteomic Core at The Fred Hutchinson Cancer Research Center, Seattle, WA. Samples were further desalted in-line using a reversed-phase trap column (100 μm × 20 mm) packed with Magic C_18_AQ (5 μm 200 Å resin; Michrom Bioresources) and peptides were separated on a reversed phase column directly mounted to the electrospray ion source [75 μm × 250 mm column packed with Magic C_18_AQ resin (5 μm 200 Å resin; Michrom Bioresources)]. Chromatographic separations were performed at a flow rate of 400 nL/min to apply a 90 min gradient of 7% to 35% ACN in 0.1% formic acid. The capillary temperature was set to 300 °C, and a 2750 V spray voltage was applied to the electrospray tip. The Orbitrap Elite instrument’s data dependent mode was used to switch automatically between MS survey scans [automatic gain control (AGC) target value 1,000,000; resolution 120,000; and injection time 250 msec] and MS/MS spectra acquisition (AGC target value of 50,000; 15,000 resolution; and injection time 250 msec). From the Fourier-transform full scan, the 15 most intense ions were selected for fragmentation by higher-energy collisional dissociation in the higher-energy C-trap dissociation (HCD) cell. The normalized collision energy was set to 40%. Selected ions were dynamically excluded for 30 sec with a list size of 500 and exclusion mass by mass width +/- 10ppm.

### Proteomic data analysis

Data analysis was performed using Proteome Discoverer 1.4 (Thermo Scientific, San Jose, CA) and the data were searched against the *N*. *gonorrhoeae* FA1090 database (UniProt, downloaded May 11, 2017) with the common Repository of Adventitious Proteins (cRAP,http://www.thegpm.org/crap/) FASTA file. Trypsin was set as the enzyme with 2 mis-cleavages allowed. Variable modifications included TMT6Plex (+229.163 Da) on any N-Terminus, oxidation on methionine (+15.995 Da), carbamidomethyl on cysteine (+57.021 Da), and TMT6Plex on lysine (+229.163 Da). Ten ppm was set as the precursor ion tolerance, and 0.8 Da was established as the fragment ion tolerance.

### Experimental design and statistical rationale

All experiments described above were performed using CE and MVs isolated from the *N*. *gonorrhoeae* wild type and Δ*mlaA* mutant in biological duplicates. Data were searched using Sequest HT. All search results were run through Percolator for scoring. Quantification was performed using the canned TMT6plex method through Proteome Discoverer to compare the normalized total reporter ion intensity between WT and Δ*mlaA* CE or MV fractions for each biological replicate. The mass spectrometry proteomics data have been deposited to the ProteomeXchange Consortium via the PRIDE [104] partner repository with the dataset identifier PXD008673. A False Discovery Rate of 1% was applied, and only proteins identified by ≥1 unique and ≥2 total peptides with a score ≥ 1 for every detected peptide were included for further analysis. Proteins were considered differentially expressed when the calculated ratios were below 0.67 or above 1.50.

### Biofilm characterization

The ability of strains to produce biofilms was assessed using a method adapted from Anderson *et al*. [105]. Non-piliated bacteria, cultured as above, were collected from plates and suspended to OD_550_ = 1.5 in supplemented GCBL. One hundred microliters of this suspension were added to the wells of a 96-well flat-bottomed microtiter plate (Corning #3370). Water was added to all remaining wells to minimize the effects of evaporation. The plate was wrapped in plastic wrap and incubated at 37 °C in a 5% CO_2_ environment for 24 h without shaking. After incubation, samples from each well were serially diluted and spotted onto GCB plates for CFU/mL enumeration of viable planktonic bacteria. Planktonic bacteria and spent media were removed and biofilm wells were washed once with PBS. Plates were allowed to dry for ~4 h at room temperature, after which 65 μL of 0.1% crystal violet were added to each well and incubated for 15 min at room temperature. Wells were then washed 3 times with PBS and allowed to dry overnight at room temperature. Biofilms were dissolved by the addition of 125 μL 30% acetic acid, incubation at room temperature for 30 min, and shaking on a microplate vortexer. Biofilm mass was measured on a BioTek Synergy HT plate reader (BioTek) at 550 nm.

### Competitive infection of the murine lower genital tract

Female BALB/c mice (6 to 8 weeks old; Charles River Laboratories Inc., Wilmington, MA; NCI Frederick strain of inbred BALB/cAnNCr mice, strain code 555) were treated with 0.5 mg of Premarin given two days prior to, the day of, and two days after bacterial inoculation to increase susceptibility to *N*. *gonorrhoeae*. Mice were also given antibiotics to suppress the overgrowth of commensal flora that occurs under the influence of estrogen [106]. Groups of mice were inoculated vaginally with similar numbers of WT FA1090 and either isogenic Δ*mlaA or* **Δ***mlaA*/P_lac_::*mlaA* bacteria (total dose 10^6^ CFU; 7 mice/group). Vaginal swabs were collected on days 1, 3, and 5 post-inoculation and suspended in 100 μL GCBL. Swab suspensions and inocula were cultured quantitatively on GCB supplemented with streptomycin (total number of CFUs) and GCB with streptomycin and kanamycin (Δ*mlaA*, **Δ***mlaA*/P_lac_::*mlaA*; CFU). Results are expressed as the competitive index (CI) using the equation CI = [mutant CFU (output)/wild-type CFU (output)]/[mutant CFU (input)/wild-type CFU (input)]. The limit of detection of 1 CFU was assigned for a strain that was not recovered from an infected mouse. A CI of >1 indicates that the mutant is more fit than the WT strain.

### Bioinformatic analyses

The MlaA amino acid sequence was used to search the non-redundant NCBI protein database to search for homologous proteins, and the ClustalOmega online tool was used to align sequences to generate a distance matrix. Signal peptides recognized by Signal Peptidase I or II were predicted with the SignalP 4.1 server (http://www.cbs.dtu.dk/services/SignalP/) or the LipoP 1.0 server (http://www.cbs.dtu.dk/services/LipoP/). MEGA7 software was employed to align sequences with the ClustalW tool for examination of phylogenetic relationships. Subsequently, a maximum likelihood tree was generated in MEGA using the Jones-Taylor-Thornton model to calculate a pairwise distance matrix [107]. To generate the initial tree, Neighbor-Join and BioNJ algorithms were applied to the matrix, and the tree was heuristically searched with the Nearest-Neighbor-Interchange method. Five hundred bootstrap replicates were applied to test the phylogenies, and the lowest log-likelihood tree is presented. The *mlaA* nucleic acid sequence was used to query the *Neisseria* Multilocus Sequence typing Database for SNP analysis of the locus (NEIS1933) across the 44,289 isolates deposited as of December 2, 2017. Phylogenetic analyses of MlaA among *N*. *gonorrhoeae* and between all *Neisseria* isolates were performed as above. Amino acid sequences of proteins identified through our quantitative proteomic investigations were used to query the NCBI non-redundant protein database using BLASTp (https://blast.ncbi.nlm.nih.gov/Blast.cgi?PAGE=Proteins) to gain insights into their functions.

### Statistical analyses

All statistical analyses were performed using GraphPad Prism software (version 6.0h for Mac OS X) with the exception of proteomic data and animal studies described above. Built-in two-way ANOVA (using Sidak’s multiple comparisons test) or unpaired *t*-test analyses were used to test for statistical significance at *p*<0.05.

### Ethics statement

Animal experiments were conducted at the Uniformed Services University of the Health Sciences (USUHS) according to the guidelines of the Association for the Assessment and Accreditation of Laboratory Animal Care under protocol no. MIC16-488 that was approved by the University’s Institutional Animal Care and Use Committee. The USUHS animal facilities meet the housing service and surgical standards set forth in the “Guide for the Care and Use of Laboratory Animals” NIH Publication No. 85–23, and the USU Instruction No. 3203, “Use and Care of Laboratory Animals”. Animals are maintained under the supervision of a full-time veterinarian. For all experiments, mice were humanely euthanized by trained personnel upon reaching the study endpoint using a compressed CO_2_ gas cylinder in LAM as per the Uniformed Services University (USU) euthanasia guidelines (IACUC policy 13), which follow those established by the 2013 American Veterinary Medical Association Panel on Euthanasia (https://www.usuhs.edu/mps/facilities-resources).

Supplemental Materials and Methods are described in S1 Text.

## Supporting information

S1 Fig Phylogenetic relationships between *Neisseria* MlaA alleles. Phylogenetic trees of MlaA alleles were constructed for alleles found in all *Neisseria* isolates (A) and among *N*. *gonorrhoeae* (B). Maximum likelihood trees were generated in MEGA7 using the Jones-Taylor-Thornton method. FA1090 MlaA allele (allele 42) is boxed in red for each tree.
(TIF)

S2 Fig Local genome context of MlaA homologs. *N*. *gonorrhoeae* FA1090 *mlaA* was used to query the BioCyc database (biocyc.org) to align *mlaA* homologs in the genomes of the bacterial strains shown. MlaA is shown in yellow hashed boxes in the top panel and in purple hashed boxes in the bottom panel. Orthologous proteins are the same color in each panel.
(EPS)

S3 Fig Purification of MBP-MlaA fusion protein. (A) MBP-MlaA was purified by affinity chromatography with a MBPTrap column. The MBP tag was subsequently cleaved by overnight incubation with TEV protease, and the cleaved protein products were subjected to nickel affinity chromatography. Elution fractions were analyzed by SDS-PAGE and coomassie staining. (B) Nickel column fractions were pooled, concentrated, and subjected to size exclusion chromatography. Elutions were separated by SDS-PAGE and silver stained. Migration of molecular mass markers (in kDa) is indicated on the left. Major protein bands are marked by arrows. MBP, maltose binding protein; TEV, Tobacco Etch Virus; SDS-PAGE, sodium dodecyl sulfate-polyacrylamide gel electrophoresis.
(EPS)

S4 Fig MlaA induction testing in two *N*. *gonorrhoeae* strain backgrounds and in *E*. *coli* harboring complementation plasmid. (A) Five complementation strains were constructed in the FA1090 and WHO X strain backgrounds, as indicated. WT and isogenic Δ*mlaA* knockouts in FA1090 or WHO X, as well as all complementation strains, were cultured aerobically in GCBL in the absence or presence of the indicated concentrations of IPTG until mid-logarithmic growth (OD_600_ of 0.6–0.8) and collected for immunoblot analysis. Equivalent OD_600_ units were separated by SDS-PAGE, transferred to nitrocellulose membranes, and probed with anti-MlaA antiserum. MlaA is indicated by an arrow, and a non-specific cross-reactive band observed in the FA1090 strain is indicated with an asterisk. (B) Coomassie stained gel of samples presented in Fig 3E in the main text, acting as a loading control. GCBL, gonococcal base liquid medium; IPTG, isopropyl β-D-thiogalactopyranoside; OD_600_, optical density at 600 nm; SDS-PAGE, sodium dodecyl sulfate-polyacrylamide gel electrophoresis.
(EPS)

S5 Fig Assessments of Δ*fur*/P_lac_::*fur* strain. (A) Demonstration of Fur’s essential role in FA1090 viability. Δ*fur*/P_lac_::*fur* was plated on GCB supplemented with (bottom plate) or without (upper plate) 0.1 mM IPTG. Robust growth was observed only in the presence of IPTG. (B) WT FA1090, isogenic knockout Δ*mlaA*, and conditional knockout Δ*fur*/P_lac_::*fur* were cultured for 6 h in the presence (Fe-) or absence (SGC) of 25 μM desferal. Fur expression was induced by the addition of 10, 50, or 100 μM IPTG. Bacterial growth was monitored every hour by OD_600_ measurement. Mean ± SEM is presented, *n* = 3. Timepoints at which all strains’ OD_600_ values under iron starvation were significantly different from WT under iron repletion are indicated with an asterisk, *p*<0.05. (C) TbpB immunoblot as presented in Fig 5I, with the exception that the blot was overexposed to demonstrate low TbpB expression levels in WT under SGC and in Δ*fur*/P_lac_::*fur* under SGC with increasing Fur induction. GCB, gonococcal base medium; IPTG, isopropyl β-D-thiogalactopyranoside; OD_600_, optical density at 600 nm; SGC, standard growth conditions; SEM, standard error of the mean.
(EPS)

S6 Fig Assessment of viable planktonic cells during biofilm growth. Suspensions of WT FA1090 and isogenic knockout Δ*mlaA* bacteria standardized to an OD_600_ of 1.5 in GCBL were cultured in 96 well plates for 24 h in 5% CO_2_ at 37°C. Planktonic bacteria were removed prior to biofilm processing, serially diluted, and spotted onto GCB for CFU/mL enumeration. Mean ± SEM is presented for 12 biological replicates, each with 3 or 4 technical replicates, for a total of 46 datapoints. GCBL, gonococcal base liquid medium; GCB, gonococcal base medium; OD_600_, optical density at 600 nm.
(EPS)

S7 Fig Competitive infection of complementation strain in the murine gonorrhea model. Female BALB/c mice were inoculated intravaginally with approximately equal numbers of CFUs of WT and Δ*mlaA*/P_lac_::*mlaA* bacteria (~10^6^ CFU total *N*. *gonorrhoeae*; 7 mice per group). Vaginal swabs taken on days 1, 3, and 5 post-infection were cultured for CFU/mL enumeration on solid media containing streptomycin (total bacteria) or media containing streptomycin and kanamycin (Δ*mlaA*/P_lac_::*mlaA* bacteria). Experiments were repeated three times and results are expressed as the geometric mean of the competitive index (CI): [mutant CFU (output) / WT CFU (output)] / [mutant CFU (input) / WT CFU (input)]. A CI > 1 indicates that the mutant was more fit during the competition. A value of 1 CFU was assigned for any strain not recovered from an infected mouse. CFU, colony forming unit.
(EPS)

S1 Text Supporting information. Table 1 in S1 Text: Amino acid identity of members of the *N*. *gonorrhoeae* Mla operon with their *E*. *coli* homologs. Table 2 in S1 Text: Agar dilution assessment of WT, Δ*mlaA*, Δ*mlaA*/P_lac_::*mlaA*, and Δ*mlaA*/P_lac_::*pldA* MICs.
(DOCX)

S1 File Clustal Omega distance matrix of *N*. *gonorrhoeae* MlaA homolog amino acid identities. (XLSX)

S2 File Quantitative proteomics results of WT and Δ*mlaA* cell envelopes. (XLSX)

S3 File Quantitative proteomics results of WT and Δ*mlaA* membrane vesicles. (XLSX)
