# Supplementary figures and images for: Neisseria gonorrhoeae MlaA influences gonococcal virulence and membrane vesicle production
Source: PLoS Pathog. 2019 Mar 7;15(3):e1007385. doi: 10.1371/journal.ppat.1007385 (PMC6424457; doi:10.1371/journal.ppat.1007385)

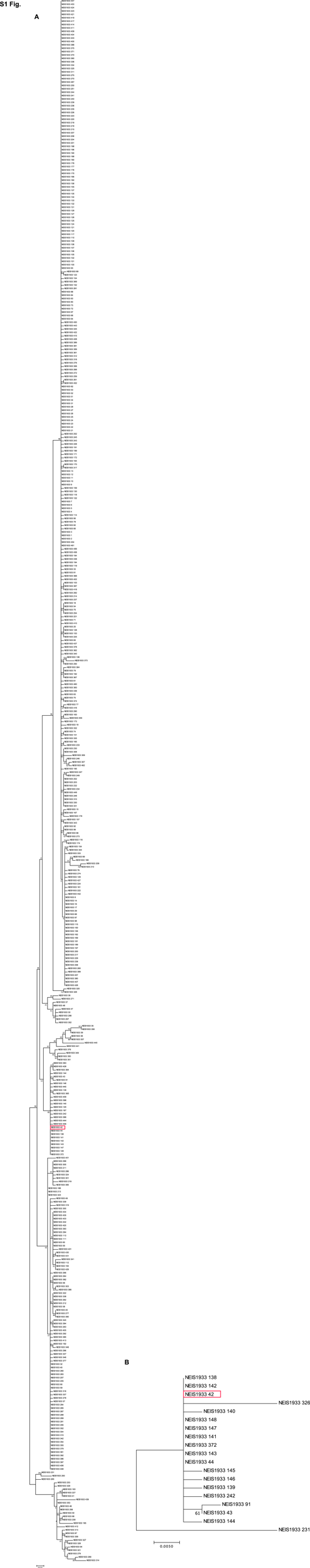

Supplement: S1 Fig — Phylogenetic trees of MlaA alleles were constructed for alleles found in all Neisseria isolates (A) and among N. gonorrhoeae (B). Maximum likelihood trees were generated in MEGA7 using the Jones-Taylor-Thornton method. FA1090 MlaA allele (allele 42) is boxed in red for each tree. (TIF) [file ppat.1007385.s001.tif]
