# Supplementary material for: Neisseria gonorrhoeae MlaA influences gonococcal virulence and membrane vesicle production
Source: PLoS Pathog. 2019 Mar 7;15(3):e1007385. doi: 10.1371/journal.ppat.1007385 (PMC6424457; doi:10.1371/journal.ppat.1007385)
Supplement: S1 Text — Table 1 in S1 Text: Amino acid identity of members of the N. gonorrhoeae Mla operon with their E. coli homologs. Table 2 in S1 Text: Agar dilution assessment of WT, ΔmlaA, ΔmlaA/Plac::mlaA, and ΔmlaA/Plac::pldA MICs. (DOCX) [file ppat.1007385.s008.docx]

**SUPPORTING INFORMATION**

**SUPPLEMENTAL MATERIALS AND METHODS**

**DNA manipulations.** MlaA lacking the signal peptide was amplified from purified FA1090 genomic DNA using primers MlaA-MBP-F, GATCCCATGGAGGATCTGTACTTTCAGAGCGAAACCCGCCCCGCCGAC, and MlaA-MBP-R, GATCAAGCTTGCTTTAGTGGTGATGGTGATGATGGGGTTGTGTTCCAGGTTGCGTTTCGG, digested with NcoI and HinDIII (New England Biolabs; recognition sites underlined), and cloned into similarly-digested pMBP-27b. Cloning resulted in the maltose binding protein (MBP) fused to the N-terminus of MlaA with two intervening TEV recognition. The MlaA C-terminus was also tagged with a 6 × Histidine tag. The resulting construct (pMBP-27b-MlaA-His) was transformed into ER2566 *E. coli* for protein production.

**Protein production and purification.** Overnight cultures of ER2566 *E. coli* harboring pMBP-27b-MlaA-His were diluted 1:100 into 1.5L of Luria-Bertani liquid media (LB) supplemented with 50 µg/mL kanamycin and cultured at 37 ºC with shaking (220 rpm). Protein production was induced by the addition of 1 mM isopropyl β-D-1-thiogalactopyranoside (IPTG) when cultures reached an OD_600_ of approximately 0.5. Induction was performed for 3 h at 37 ºC with shaking. Bacteria were collected by low-speed centrifugation (6000 × g for 10 min at 4 ºC). Pellets were subsequently suspended in lysis buffer (20 mM Tris, pH 7.4; 200 mM NaCl; 1 mM ethylenediaminetetraacetic acid [EDTA]; and a Pierce protease inhibitor tablet [ThermoFisher Scientific]), lysed by 5 passages through a French pressure cell at ~12,000 psi, and centrifuged to remove intact bacteria and cell debris. Supernatants were subjected to affinity chromatography with a MBPTrap column (GE Healthcare Life Sciences) using a Bio-Rad NGC Scout system, and protein was eluted with elution buffer (20 mM Tris, pH 7.4; 200 mM NaCl; 1 mM EDTA; 10 mM maltose). The MBP tag was cleaved by overnight incubation with a 1:40 TEV:protein (v/v) ratio during dialysis into 50 mM Tris, pH 8 and 1 mM dithiothreitol (DTT). The protein solution was subsequently applied to a Bio-Rad Bio-Scale Mini Nuvia IMAC cartridge (Bio-Rad) using the Bio-Rad NGC Scout system and eluted with nickel elution buffer (20 mM Tris, pH 8; 500 mM NaCl; 250 mM imidazole). Elutions were pooled, concentrated, and applied to a HiLoad Superdex 75 column (GE Healthcare Life Sciences) for size exclusion chromatography on the Bio-Rad NGC Scout system in column buffer (20 mM Tris, pH 8; 500 mM NaCl; 10% glycerol [v/v]). Fractions were analyzed for protein content throughout the purification process by sodium dodecyl sulfate-polyacrylamide gel electrophoresis and visualization by either colloidal coomassie G-250 staining or with the SilverQuest Silver Staining Kit (ThermoFisher Scientific).

**Agar dilution assessment of antibiotic minimal inhibitory concentrations.** Non-piliated colonies cultured as in the main text were suspended in GCBL to a density of 1 × 10^5^ CFU/mL. One hundred microliters of this suspension, corresponding to approximately 1 × 10^4^ CFU, were spread onto GCB plates supplemented with 2-fold dilutions of antibiotics indicated in the text and 0.5 mM IPTG to induce expression of MlaA or PldA. MICs were assessed after ~22 h of incubation at 37 ºC in a 5% CO_2_ atmosphere. Experiments were performed on five independent occasions and majority values are reported.

**Fur induction and iron limitation growth curves.** Non-piliated colonies of WT FA1090, isogenic knockout ∆*mlaA*, and conditional knockout ∆*fur*/P_lac_::*fur* were suspended to an OD_600_ of 0.1 in GCBL supplemented with Kellogg’s supplement I and 0.042% sodium bicarbonate, but lacking Kellogg’s supplement II. Strains were cultured for an initial 3 h period at 37 ºC with shaking (220 rpm). Fur expression was induced by the addition of 100 µM IPTG during initial growth. After 3 h, ∆*fur*/P_lac_::*fur* cultures were centrifuged at 5000 × *g* for 5 min, the supernatant was decanted to remove IPTG, and the pellet was resuspended in medium supplemented as above but lacking IPTG. All cultures were back diluted to an OD_600_ of 0.1 and supplemented with either Kellogg’s supplement II (1:1,000) or 25 µM desferal. Fur expression was induced by the addition of 10, 50, or 100 µM IPTG both under standard growth conditions and iron starvation. Bacterial growth was monitored each hour by OD_600_ measurement. Growth curves from three independent experiments were plotted in GraphPad Prism and analyzed using the built-in two-way ANOVA to test for statistical significance at *p*<0.05 using Sidak’s multiple comparisons test.

**SUPPLEMENTAL TABLES**

**Table 1. Amino acid identity of members of the *N. gonorrhoeae* Mla operon with their *E. coli* homologs.**

| **FA1090 Genetic Locus** | ***E. coli* homolog with UniProt ID** | **Amino Acid Identity^a^** |
| --- | --- | --- |
| *ngo2120* | *mlaB* (P64602) | 24.3% |
| *ngo2119* | *mlaC* (P0ADV7) | 24.8% |
| *ngo2118* | mlaD (P64604) | 37.2% |
| *ngo2117* | mlaE (P64606) | 52.7% |
| *ngo2116* | mlaF (P63386) | 46.5% |

^a^Amino acid identity was assessed with the EMBOSS Needle online pairwise protein alignment tool (https://www.ebi.ac.uk/Tools/psa/emboss_needle/).

**Table 2. Agar dilution assessment of WT, ∆*mlaA*, ∆*mlaA*/P_lac_::*mlaA*, and ∆*mlaA*/P_lac_::*pldA* MICs.**

|  | **Polymyxin B^a^** | **Vancomycin^a^** | **Ampicillin^a^** |
| --- | --- | --- | --- |
| **WT** | 128 | 16 | 0.25 |
| **∆*mlaA*** | 64 | 8 | 0.25 |
| **∆*mlaA*/P_lac_::*mlaA*** | 128 | 8 | 0.25 |
| **∆*mlaA*/P_lac_::*pldA*** | 32 | 8 | 0.25 |

^a^MIC values are presented in µg/mL
